# Prevalence, outcomes, and predictive factors: a systematic literature review to inform the development of EULAR Points to Consider for the definition of Difficult-to-Manage and Treatment-Refractory psoriatic arthritis

**DOI:** 10.1016/j.ero.2025.07.007

**Published:** 2025-10-30

**Authors:** Stephanie R. Harrison, George E. Fragoulis, Xabier Michelena, Cristina Macía-Villa, Louise Falzon, Stefan Siebert, Helena Marzo-Ortega, Alexandre Sepriano, Pedro M. Machado

**Affiliations:** 1 National Institute for Health and Care Research (NIHR) Leeds Biomedical Research Centre (BRC), Chapel Allerton Hospital, The Leeds Teaching Hospitals NHS Trust, Leeds, UK; 2 Leeds Institutes of Cardiovascular and Metabolic Medicine (LICAMM) and Data Analytics (LIDA), University of Leeds, Leeds, UK; 3 Joint Academic Rheumatology Program, First Department of Propaedeutic and Internal Medicine, Athens, Greece; 4 Rheumatology Unit, Digitalization for the Sustainability of the Healthcare System (DS3) and Vall d’Hebron Hospital Universitari, Vall d’Hebron Barcelona Hospital Campus, Barcelona, Spain; 5 Rheumatology Department, Ramón y Cajal University Hospital, Madrid, Spain; 6 School of Medicine and Population Health, University of Sheffield, Sheffield, UK; 7 School of Infection and Immunity, University of Glasgow, Glasgow, UK; 8 NOVA Medical School, Universidade NOVA de Lisboa, Lisboa, Portugal; 9 Department of Rheumatology, Leiden University Medical Centre, Leiden, The Netherlands; 10 Department of Neuromuscular Diseases, University College London Queen Square Institute of Neurology, University College London, London, UK; 11 Department of Rheumatology, Northwick Park Hospital, London North West University Healthcare NHS Trust, London, UK; 12 National Institute for Health Research (NIHR) University College London Hospitals Biomedical Research Centre, University College London Hospitals NHS Foundation Trust, London, UK

## Abstract

**Objectives:**

To perform a systematic literature review (SLR) to inform the EULAR Task Force “Points to Consider for the definitions of Difficult-to-Manage (D2M) and Treatment-Refractory (TR) psoriatic arthritis (PsA)”.

**Methods:**

The SLR addressed 3 separate questions concerning the following: (1) prevalence, (2) outcomes, and (3) predictors of D2M/TR PsA. A search was conducted in MEDLINE (Ovid), Cochrane Database of Systematic Reviews, CENTRAL, EMBASE, and Epistemonikos from inception to March 3, 2024, as well as EULAR/ACR abstracts for 2023/24 and 2022/23, respectively. This systematic review adhered to Preferred Reporting Items for Systematic Reviews and Meta-Analyses guidelines and was registered on PROSPERO. Data are summarised descriptively. Meta-analysis was not possible due to significant heterogeneity and/or lack of high-quality evidence. Risk of bias (RoB) was assessed using the Newcastle-Ottawa Scale.

**Results:**

Overall, 70, 4, and 25 articles/abstracts were included for questions 1, 2, and 3, respectively. For question 1, 30/70 records had low/moderate RoB, with the prevalence of D2T PsA ranging from 5% to 80% depending on both the timepoint at which prevalence was measured and the definition of D2M/TR PsA applied, which varied significantly between studies. For question 2, all records had a high RoB; therefore, no conclusions could be drawn. For question 3, all records had a moderate RoB, and 84 categories of potential predictors were identified, with large differences in strength and direction of association between studies.

**Conclusions:**

Despite significant heterogeneity in the published literature regarding the scope, definition, long-term outcomes, and predictors of D2M/TR PsA, certain patterns emerged that helped shape the final EULAR Task Force recommendations.

## INTRODUCTION

Psoriatic arthritis (PsA) is an inflammatory arthritis affecting approximately 0.1% to 0.5% of the general population and up to 30% of patients with psoriasis [1]. In addition to psoriasis and musculoskeletal (MSK) manifestations (peripheral arthritis, dactylitis, enthesitis, and axial involvement), which characterise the condition, PsA can have additional extra-MSK manifestations (EMMs) such as uveitis and inflammatory bowel disease (IBD). It is also associated with several comorbidities, including cardiometabolic diseases, depression/anxiety, secondary osteoarthritis, and pain syndromes [2]. Recognizing the heterogeneity and the multifaceted nature of the disease, the inclusive term ‘psoriatic disease’ has been suggested to encompass these aspects [3].

Over the past decade, significant advances in the understanding of PsA pathophysiology [4] have led to the development of several targeted therapies, including IL-23, IL-17, and JAK inhibitors. However, achieving low disease activity (LDA) or minimal disease activity (MDA) remains challenging. Indeed, 2 independent systematic review/meta-analyses found that regardless of whether LDA or MDA was used, one third of patients still failed to meet the target [5,6]. Possible reasons include the following: (a) comorbidities (eg, cardiometabolic diseases and malignancies), which can limit the use of certain drugs and may have a confounding effect on the patient-reported outcome measures (PROMs) used to monitor treatment responses [7,8], (b) health inequities and disparities in access to health care in different healthcare systems and countries [9], and (c) the diverse immunologic mechanisms driving the underlying disease biology, and mechanisms of nonresponse, which may vary depending on diseases manifestations and affected tissues [10]. A recently published review by Witte et al. [11] explores clinical, imaging, and immunologic diversity of PsA in detail and outlines how recent advances in imaging and therapeutic strategies in PsA may contribute to precision medicine and improved patient outcomes in the future. Moving forward, further research in this area is essential for improving both the quality of life and treatment outcomes for all patients with PsA.

Similar challenges in achieving optimal treatment outcomes have been recognized in rheumatoid arthritis (RA) and axial spondyloarthritis (axSpA), leading to the establishment of the ‘difficult-to-treat RA’ concept, endorsed by EULAR [12], and the ‘difficult-to-manage axSpA’ concept, endorsed by the Assessment of SpondyloArthritis international Society (ASAS) [13]. No equivalent definition currently exists for PsA, which represents a clear unmet need both in research and clinical practice [14,15]. Furthermore, understanding the evidence base for the same concept in PsA can be challenging. Many different terminologies are used in the literature, including Difficult-to-Treat (D2T), Difficult-to-Manage (D2M), and Treatment-Refractory (TR) PsA [14]. Often, the same term(s) are used interchangeably, and sometimes the same terms are used to describe subtly different concepts, making it difficult to interpret the published literature. Further, there is appreciable heterogeneity and complexity of clinical manifestations contributing to unsatisfactory treatment response in PsA, including both biological and nonbiological drivers [14].

This systematic literature review (SLR) was performed to inform the formulation of EULAR PtC for the definition of the D2T/D2M/TR PsA concept and aimed to investigate the prevalence, long-term outcomes, and predictive factors for D2M/TR PsA. Hereafter, only the terms D2M/TR will be used as this is the recommended terminology suggested by the EULAR TF in their final PtCs and definitions (manuscript submitted) to ensure consistency for all future use and research in this area. This preferred terminology, referring to D2M/TR, incorporates all previously published related concepts such as D2T.

## METHODS

Following the initial meeting of the EULAR TF to define the scope of the TF and this SLR, the TF Steering Committee convened and formulated the SLR research questions, as agreed upon by the TF members.

The SLR was then conducted by the fellows and supervised by the methodologists. This SLR adheres to best practice for SLRs as outlined in the Cochrane Handbook, is in accordance with the Preferred Reporting Items for Systematic Reviews and Meta-Analyses (PRISMA) guidelines [16,17] and was registered prospectively on PROSPERO (CRD42024558589; approved July 8, 2024) [18].

The following 3 research questions were discussed and approved by the TF:

Q1: What is the prevalence of D2M/TR disease, treatment discontinuation of one or more biologic/targeted synthetic disease-modifying antirheumatic drugs (b/tsDMARDs), or related concepts, in real-world evidence for PsA?

Q2: What are the long-term impacts and outcomes of D2M/TR disease, treatment discontinuation, or related concepts in PsA?

Q3: What are the predictive factors of D2M/TR disease, treatment discontinuation, or related concepts in PsA?

These questions were framed and structured according to the updated EULAR standardised operating procedures [19,20] using the ‘Patients, Intervention, Comparator or Control, Outcome, Type of study’ (PICO) format [21]. The PICO format for each of the above questions is shown in Table 1. Of note, our approach was highly inclusive and broad, designed to capture a wide spectrum of concepts related to D2T, D2M, resistant, and refractory disease, as agreed by the TF members.

**Table 1:** PICOs for the systematic literature review research questions.

**Q1: What is the prevalence of difficult-to-treat/difficult-to-manage/resistant/refractory disease, treatment discontinuation of one or more b/tsDMARDs, or related concepts in real-world evidence for PsA?**	

Patient	Patients with PsA (physician diagnosis, may or may not meet CASPAR criteria)
Intervention	bDMARD, biologics, tsDMARDs, targeted synthetic DMARDs, names of individual drugs (brand and trade names, biosimilars)
Comparator	No comparator
Outcome	Difficult-to-treat, D2T, difficult-to-manage, D2M, true refractory, refractory, resistant, treatment discontinuation, side effects, intolerance, persistent disease activity, high disease activity, primary nonresponse, secondary nonresponse, nonresponse, treatment failure, switching, inadequate response.

**Q2: What are the long-term impacts and outcomes of difficult-to-treat/difficult-to-manage/resistant/refractory disease, treatment discontinuation, or related concepts in PsA?**	

Patient	Patients with PsA (physician diagnosis, may or may not meet CASPAR criteria)
Intervention	Difficult-to-treat, D2T, difficult-to-manage, D2M, true refractory, refractory, resistant, treatment discontinuation, side effects, intolerance, persistent disease activity, high disease activity, primary nonresponse, secondary nonresponse, nonresponse, treatment failure, switching, inadequate response.
Comparator	Not meeting the definition of D2M/D2T/resistant/treatment discontinuation
Outcomes	Quality of life, work, structural progression, multi-tissue involvement (skin, joints, pain), phenotypes, ‘domains’, disease outcome measures, composite, single, multimorbidities/comorbidities (OA, gout, CVD/MACE, diabetes, liver disease/MASLD/NAFLD, osteoporosis), joint replacement, extramusculoskeletal manifestations, mental health, depression/anxiety, fatigue, fibromyalgia/chronic pain syndromes, mortality, hospitalisation, cost to service provider

**Q3: What are the predictive factors of difficult-to-treat/difficult-to-manage/resistant/refractory disease, treatment discontinuation, or related concepts in PsA?**	

Patient	Patients with PsA (physician diagnosis, may or may not meet CASPAR criteria)
Intervention	Any treatment(s)/intervention(s) as defined by the authors
Comparator	No comparator
Outcome	Difficult-to-treat, D2T, difficult-to-manage, D2M, true refractory, refractory, resistant, treatment discontinuation, side effects, intolerance, persistent disease activity, high disease activity, primary nonresponse, secondary nonresponse, nonresponse, treatment failure, switching, inadequate response

b/tsDMARD, biological/ targeted synthetic disease-modifying antirheumatic drug; CASPAR, classification of psoriatic arthritis; CVD, cardiovascular disease; D2M, difficult-to-manage; D2T, difficult-to-treat; MACE, major adverse cardiovascular event; MASLD, metabolic dysfunction-associated steatotic liver disease; NAFLD, nonalcoholic fatty liver disease; OA, osteoarthritis; PsA, psoriatic arthritis; Q, research question.

### Search strategy and study selection

A single search strategy was designed by the TF fellows (GF, SRH), The Emerging EULAR Network (EMEUNET) members (XM, CMV), and the TF methodologists (PMM, AS), with support from a librarian (LF). Details of the search strategies are provided in Supplementary Table S1. The search was limited to full-text English articles from database inception to the date of the search (March 21, 2024) and run in MEDLINE (Ovid), The Cochrane Database of Systematic Reviews, CENTRAL, EMBASE, and Epistemonikos. No filters were applied, except for Epistemonikos, which was limited to SLRs. Conference abstracts from EULAR (2023 and 2024) and ACR (2022 and 2023) annual meetings were also reviewed using a search that included the words ‘PsA’ or ‘Psoriatic Arthritis’ in the title or keywords.

**Figure 1:**
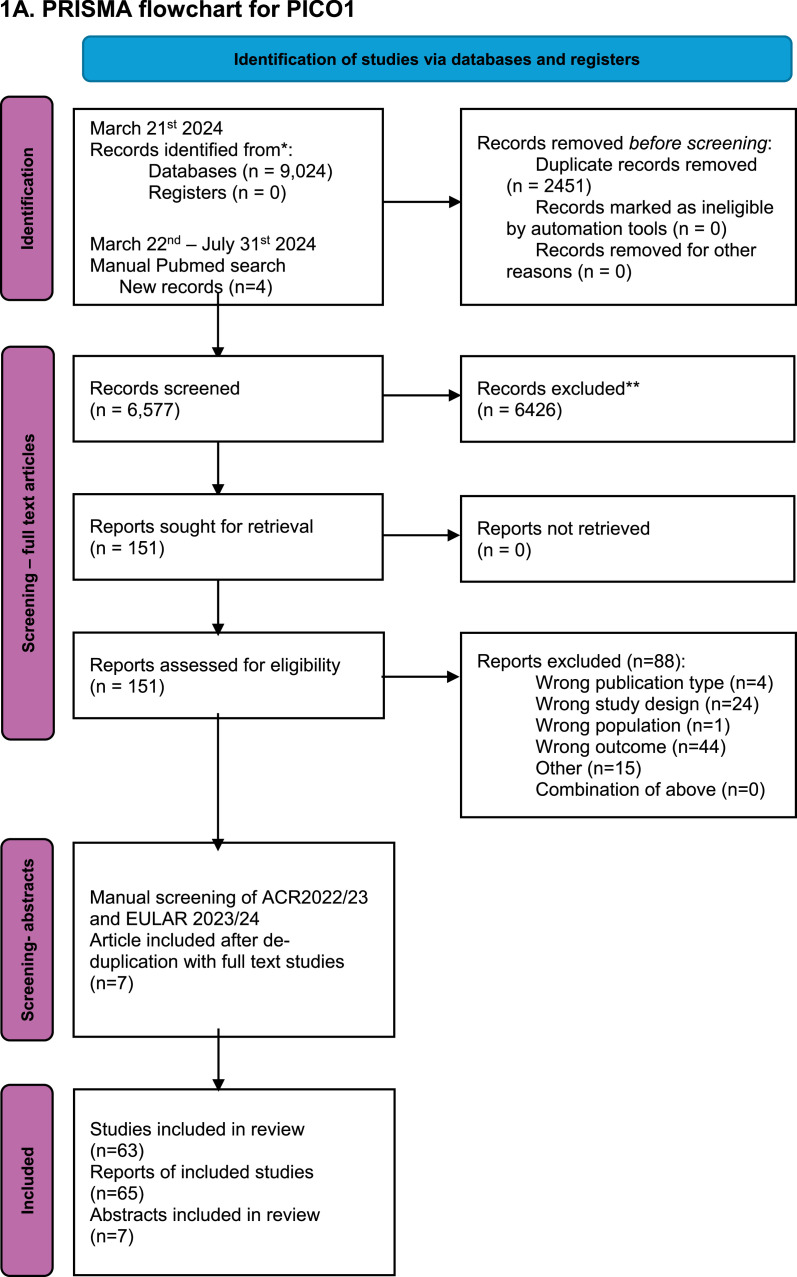

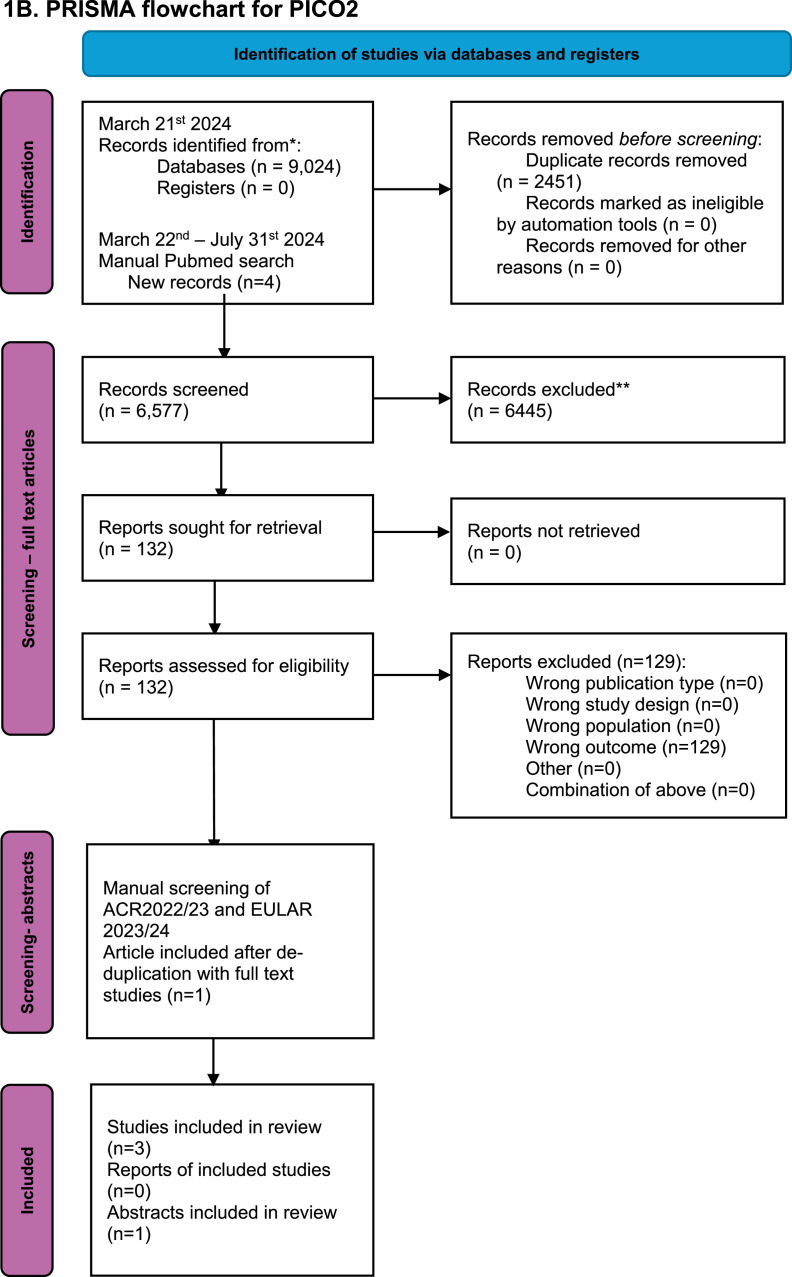

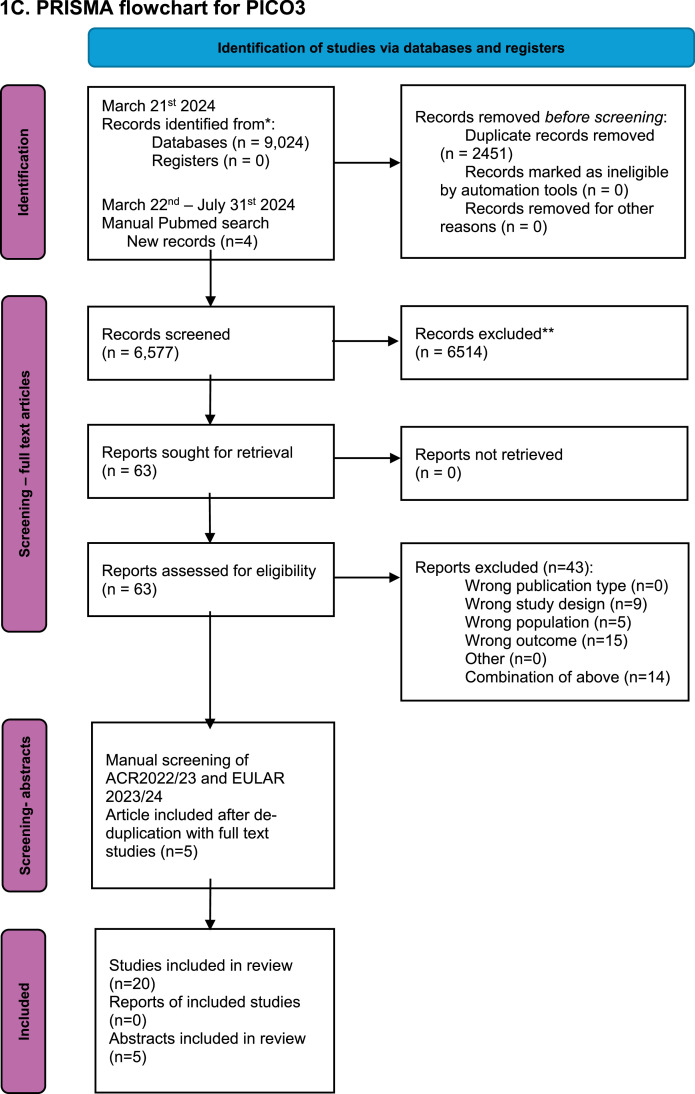
PRISMA flowcharts of article screening and exclusion for (A) PICO1, (B) PICO2, and (C) PICO3. *Reasons for exclusion at the title/abstract screening stage were not recorded individually for each search result; however, they were the same as those applied during full-text screening. PICO, Patient/Population, Intervention, Comparison, Outcome; PRISMA, Preferred Reporting Items for Systematic Reviews and Meta-Analyses.

Pairs of fellows/EMEUNET members initially screened 10% of the titles and abstracts in duplicate using Rayyan [22]. Any disagreements were resolved through discussion and, when a consensus could not be reached, the methodologists (PMM/AS) made the final decision. If agreement exceeded the predefined cutoff of 90%, the remaining 90% of articles were not screened in duplicate. If there was uncertainty, the article was discussed with the other fellow and/or methodologists to reach a consensus. Study selection was made separately for each PICO/research question. The number of articles included/excluded at each stage is shown in Figure 1.

### Risk of bias (quality) assessment

Risk of bias was assessed using the Newcastle-Ottawa Scale (NOS) for case-control or cohort studies, as applicable [23]. As there is no single standardised reporting scale for NOS, we used a previously proposed scale that classifies scores of 1 to 3 as high risk of bias, 4 to 6 as moderate risk, and 7 to 9 as low risk [24].

### Data extraction and synthesis

For data extraction, a bespoke data proforma was developed for each PICO. This was piloted on the first 10% articles in duplicate by 2 fellows to ensure reproducibility and to allow for minor modifications to be made, if needed, to ensure efficiency in extracting all relevant data. The proforma demonstrated good usability and reproducibility for all 3 PICOs/SLR questions, with 100% agreement between fellows for the first 10% of articles screened. Therefore, for the remaining 90% of articles, data extraction was conducted by a single fellow for each SLR question; therefore, if an article was included in 2 SLR questions, it underwent separate data extraction by separate fellows for each specific question against which it was being assessed. In the case of uncertainty, this was resolved by discussion between fellows in the first instance and/or involvement of a methodologist if a consensus could not be reached.

Numeric data were extracted to enable comparison between studies, including descriptive statistics for all 3 PICOs (mean/median and SD/IQR for continuous data and percentage/frequencies for categorical data). Additionally, odds ratios, relative risk, or hazard ratios, and their 95% CIs, were extracted for PICOs/questions 2 and 3. Pooled/statistical meta-analyses could not be conducted due to significant differences in the types of statistical analyses and reporting of results across different studies.

## RESULTS

The literature search retrieved 6440 articles. After initial title and abstract review, 158, 4, and 67 articles were put forward for full-text review for questions 1, 2, and 3, respectively. The final number of studies that fulfilled inclusion criteria, and therefore underwent data extraction, was 70 for question 1 (63 manuscripts and 7 abstracts), 4 for question 2 (3 manuscripts and 1 abstract), and 25 for question 3 (20 manuscripts and 5 abstracts), with some studies included in multiple PICOs.

This section provides a concise summary of each PICO, aiming to present the data in a way that highlights how the SLR informed the work of the EULAR Task Force for which it was conceptualised. Further details on terminologies used, baseline demographics, and reported outcomes pertaining to each primary research study can be found in Supplementary Tables.

### PICO 1

#### Q1: What is the prevalence of D2M/TR disease, treatment discontinuation of one or more b/tsDMARDs, or related concepts in real-world evidence for PsA?

Data were extracted from 63 full-text articles and 7 abstracts for PICO1 (Fig 1 and Supplementary Table S2) [[25], [26], [27], [28], [29], [30], [31], [32], [33], [34], [35], [36], [37], [38], [39], [40], [41], [42], [43], [44], [45], [46], [47], [48], [49], [50], [51], [52], [53], [54], [55], [56], [57], [58], [59], [60], [61], [62], [63], [64], [65], [66], [67], [68], [69], [70], [71], [72], [73], [74], [75], [76], [77], [78], [79], [80], [81], [82], [83], [84], [85], [86], [87], [88], [89], [90], [91], [92], [93], [94], [95]]. Overall, 30/70 studies were assessed as having low risk of bias, 36/70 were moderate risk, and 7/70 were high risk according to the NOS.

Baseline patient characteristics varied between studies. Different measures of central tendency and variability/dispersion were used; hence, statistical meta-analysis was not possible, and herein, a descriptive summary of baseline characteristics is provided. The range of the mean values for age, body mass index (BMI), and disease duration was 44.8 to 56.0 years (available for *n* = 53/70 studies), 25.5 to 32.6 kg/m^2^ (*n* = 21/70 studies), and 5.2 to 17 years (available for *n* = 35/70), respectively. The overall ratio of females:males was approximately 50% in most studies, although the prevalence of females was higher in ∼66% studies. The overall prevalence of EMMs ranged between 1.4% to 8.9% (available for *n* = 5/70) and 0.5% to 9.8% (available for *n* = 6/70) for IBD and uveitis, respectively. Other baseline characteristics, including medical history, family history, examination findings, blood tests, and disease activity scores, were reported inconsistently between studies (Supplementary Table S3).

Various definitions were used for D2M/TR PsA. Seven studies, including 2 abstracts (43% and 57% with low and moderate risk of bias, respectively), examined the prevalence of D2T PsA using a definition combining failure of 2 or more b/tsDMARDs with persistent/high disease activity. Of note, for the latter, many different variations were used and reported, for example, having signs and symptoms of active MSK disease, high C-reactive protein, not achieving MDA, or at least moderate disease activity (as assessed by disease activity psoriatic arthritis [DAPSA]), among others. In these studies, the percentage of D2T PsA using these criteria ranged from 2.9% to 68.4%, which was narrowed down to 27% to 80% in studies with low risk of bias (Supplementary Tables S2 and S4).

Sixty studies, including 3 abstracts (43.3% and 45% with low and moderate risk of bias, respectively), reported the prevalence of treatment discontinuation of b/tsDMARDs (irrespective of line of treatment), with significant variation in both the definition of D2T PsA and time-to-event. The prevalence of D2T PsA, defined as either treatment discontinuation or switching, depending on the study, was measured at several different timepoints by different authors. Most studies, however, reported the outcome at 1 or more of 3, 6, 12, and 24 months with prevalence rates of D2T PsA of 1% to 22%, 2% to 51%, 3% to 74%, and 40% to 80%, respectively (Table 2). These percentages were similar across b/tsDMARDs drug categories. It should be noted that the frequency of treatment discontinuation/switching was lower for studies reporting outcomes for patients who were b/tsDMARDs-naïve (Supplementary Tables S5 and S6).

**Table 2:** Summary of the prevalence of D2M/TR psoriatic arthritis in real-world studies

	Criteria used to define D2M/TR			
	Failed 1 b/tsDMARDs		Persistent disease activity	Increased disease activity despite 2 b/tsDMARDs
	Treatment-naïve	Treatment-IR		
**Number of studies (number of abstracts included)**	8 (0)	52 (3)	15 (2)	7 (2)
**Prospective/retrospective (%)**	25/75	27/73	40/60	14/86
**RoB %** **(low/moderate/high)**	13/62/25	48/42/10	33/60/7	43/57/0
**Prevalence (range)**	3 mo: 1%-22% 6 mo: 2%-51% 12 mo: 3%-74% 24 mo: 27%-80%		6 mo: 57%-97% (did not achieve DAPSA remission) 12 mo: 57%-70% did not achieve MDA, 90% did not achieve VLDA	8%-34%^a^

b/tsDMARDs, biologic/targeted synthetic disease-modifying antirheumatic drugs; DAPSA, disease activity psoriatic arthritis; D2T, difficult-to-treat; D2M, difficult-to-manage; TR, treatment-refractory; IR, inadequate responders; MDA, minimal disease activity; RoB, risk of bias; VLDA, very low disease activity.

a In studies with low risk of bias.

Finally, 15 studies, including 2 abstracts (33% and 60% with low and moderate risk of bias, respectively), reported the percentage of patients with persistent or high disease activity irrespective of the line of treatment. At the 6-month timepoint, 57% to 97% of participants did not achieve DAPSA remission, and at the 12-month timepoint, 57% to 70%/90% did not achieve MDA/very low disease activity (VLDA), respectively (Table 2). No major differences were noted across different drug categories (Supplementary Tables S7 and S8).

### PICO 2

#### Q2: What are the long-term impacts and outcomes of D2M/TR disease, treatment discontinuation, or related concepts in PsA?

Only 3 publications and one congress abstract were found for the SLR based on the PICO2 question. The studies were all deemed high risk of bias using the NOS scale; therefore, any conclusions drawn from these data should be interpreted with caution. Due to the absence of high-quality studies, we present a narrative synthesis of the results to underscore both the limited evidence base and the areas where further research may be needed.

All studies also used different definitions of D2T PsA [36,[96], [97], [98]]. Outcomes measures were physical function and health status, using a variety of PROMs across all 4 studies including Health Assessment Questionnaire Disability Index (HAQ-DI), EuroQoL 5-Dimensaion 5-Level (EQ-5D-5L), Physical Component Summary Questionnaire Short Form (SF)-36, Mental Component Summary SF-36, visual analogue scale (VAS), Bristol Rheumatoid Arthritis Fatigue Multidimensional Questionnaire, Work Productivity and Activity Impairment (WPAI), Hospital Anxiety and Depression Scale, Physician Global Assessment of Disease Activity, Patient Global Assessment of Disease Activity, and Psoriatic Arthritis Impact of Disease. Outcomes were examined in univariate and/or multivariate analyses in different studies. Most outcomes were reported by only 1 of the 4 studies, except for HAQ-DI, EQ-5D-5L, and WPAI which were reported in 2 studies. The limited data and heterogeneity make it impossible to provide a summary of results; therefore, a descriptive analysis of each individual study and outcome measure is provided in Supplementary Table S9.

### PICO3

#### Q3: What are the predictive factors of D2M/TR disease, treatment discontinuation, or related concepts, in PsA?

For SLR question 3, 20 full-text articles and 5 abstracts were identified (Fig 1c) [26,42,43,48,49,56,61,71,73,83,87,90,[99], [100], [101], [102], [103], [104], [105], [106], [107], [108], [109], [110]]. All 25 studies had a moderate risk of bias according to the NOS.

With regard to study characteristics, all studies included were prospective cohort studies. The outcome of interest (D2T PsA/ relevant synonyms) was defined differently by the various study authors, including criteria such as the number of drugs, mechanisms of action, disease activity, and other factors (Supplementary Table S10).

A detailed summary of baseline characteristics as reported by study authors is available in Supplementary Table S11. Briefly, the range of mean/median values for age, BMI, and disease duration was 45.7 to 56.5 years, 26.7 to 32.6 kg/m^2^, and 7.8 to 13 years, respectively. Similar to SLR questions 1 and 2 (see previous), females were slightly more prevalent than males (∼55%-60%). As so few studies reported EMMs, and those that did so were inconsistent in the format of reporting, direct comparison between studies was not possible.

Predictors of D2T, as reported by study authors, were numerous. Among the 83 groups of predictors analysed, 39 demonstrated statistical significance (*P* < .05) or borderline significance (*P* < .10) in at least 1 univariable or multivariable analysis. The top 10 most frequently reported predictors of D2T PsA were female sex, higher swollen joint count, higher tender joint count, higher BMI, higher number of b/tsDMARDs ever used, higher number of csDMARDs ever used, higher baseline patient global VAS, concurrent skin psoriasis, higher patient pain VAS, and presence of comorbid anxiety/depression (Table 3). A detailed summary of the overall frequency and percentage positive associations for all 83 predictors is summarised in Supplementary Table S12. Additionally, the individual study results for all of the top 10 tested predictors, including effect sizes and 95% CI/*P* values, can be found in Supplementary Tables S13 to S22.

**Table 3:** Summary of top 10 tested predictors for D2M/TR PsA according to PICO3 studies.

Predictor	No. tests for this variable overall	No. of analyses that report a positive association with D2M/TR PsA (*P* < .05)		No. of analyses that report a negative association with D2M/TR PsA (*P* < .05)		Percentage of positive associations overall (%)^a^
		Multivariable statistical model	Univariable statistical model	Multivariable statistical model	Univariable statistical model	
Sex (female)	49	10	10	14	14	41.7
Age—younger age at study enrolment (years)	39	2	2	16	16	11.1
BMI (high vs low)^a^	31	5	3	11	9	28.6
csDMARD exposure^a^	26	3	5	2	13	34.8
Comorbidities—PsO (skin)^a^	23	3	8	5	1	64.7
Disease activity—dactylitis^a^	23	0	2	7	13	9.1
Clinical—SJC (higher)	21	9	4	2	5	65.0
Duration PsA (y)	20	2	4	2	12	30.0
Disease activity—enthesitis^a^	20	2	2	6	9	21.1
Clinical—TJC (higher)	19	4	6	2	6	55.6

BMI, body mass index; csDMARD, conventional synthetic disease-modifying antirheumatic drug; D2M, difficult-to-manage; D2T, difficult-to-treat; PICO, Patient/Population, Intervention, Comparison, and Outcome; No., number; PsA, psoriatic arthritis; PsO, psoriasis; SJC, swollen joint count; TJC, tender joint count; TR, treatment-refractory.

a Percentages are reported to 1 decimal place and were calculated by dividing the number of positive associations (univariate or multivariate) by the total number of tests for the variable overall.

## DISCUSSION

From the outset of this project, it was evident that the complexity of PsA contributes to significant heterogeneity used to date in the definitions to describe this subset of patients with PsA. After appraising the available literature and following a thorough and robust process including expert opinion, the TF voted on the use of the D2M and TR terminology, with TR representing a subset of the D2M group. D2M PsA refers to a broader concept that includes drivers such as inflammation, comorbidities, psychosocial factors, and other elements. It incorporates a second concept, TR PsA, defined by persistent disease activity and objective evidence of active inflammation. This approach aligns with the recently published ASAS axSpA definitions [13].

In appraising the evidence from this SLR, we adopted an inclusive approach to capture the various concepts related to D2M/TR disease, such as treatment discontinuation and the percentage of patients not achieving MDA, as reported by study authors. Influenced by the EULAR definition for D2T RA [12], most recent studies in PsA have used or adapted this terminology, basing their definitions on the number and/or mechanism of action of previously received treatments, disease activity, or combinations of both [26,72,90].

In this SLR, we found that in real-world settings, 40% to 80% people with PsA discontinue or switch treatment with b/tsDMARDs within 24 months, and that only 30% of patients achieve MDA in the first year of treatment (Table 1). These figures are in agreement with a recent SLR/meta-analysis focusing on MDA and evaluating both real-world studies and RCTs [5].

Several characteristics were associated with D2M/TR PsA. The association of D2M/TR PsA with characteristics such as female sex and high BMI is not unexpected, as it is increasingly recognized that females display poorer treatment responses than males [111,112] and that treatment outcomes (at least for TNF inhibitors) are negatively affected by increased BMI [113]. Besides, adipose tissue is not an ‘innocent bystander’; in an obese state, it is considered to contribute to the inflammatory responses [114] more broadly and specifically in relation to rheumatic and MSK diseases and PsA [115,116]. Comorbid anxiety and depression were also found by many studies to be associated with D2M/TR PsA. Mental health disorders are one of the most common comorbidities encountered in PsA [2] and have been shown to be associated with adverse treatment outcomes [8]. The relationship between these factors seems to be bidirectional, as inflammatory mediators have been found to be associated with depression in animal models and human studies in inflammatory arthritis [[117], [118], [119]].

On the other hand, there is evidence that depression and anxiety might affect patient-reported outcomes [7]. Of note, PROMs (eg, higher baseline patient global VAS and patient pain VAS) were often higher in individuals with D2M/TR PsA in our SLR.

The complex relationship between disease activity and inflammatory mechanisms is undoubtedly a key factor underlying the breadth of definitions and outcomes observed across all PICOs. Efficacy of b/ts-DMARD treatments varies between EMMs as reflected in international treatment guidelines for PsA [[120], [121], [122], [123]]. Moreover, axial disease does not respond to the same treatment as peripheral disease [[120], [121], [122], [123]]. Such variation points towards both convergence and divergence in the molecular pathways underpinning the numerous manifestations of PsA [124]. Interpatient heterogeneity also exists due to differences in each individual's immune biology, environment, genetics, and lifestyle. Although some of these factors are fixed (eg, genetics), others can change over time. Predicting in whom, and when, such risk factors may trigger onset or progression of PsA remains a significant area of unmet clinical need to facilitate precision medicine approaches to diagnosis, prognostication, and treatment response [125,126]. For example, a sexually transmitted or gastrointestinal infection in an HLA-B27 positive individual may lead to no MSK symptoms, may trigger a self-limiting inflammatory arthritis, or may progress to a chronic inflammatory arthropathy such as PsA [127]. Other factors that can modulate the immune system, and thus treatment response, include obesity (as discussed previously), mechanical or physiological stress at the joint/entheses, psychological stressors [128,129], sex [130], hormonal changes (eg, menopause) [131], and immunosenescence with aging [132,133].

Moreover, there is increasing evidence that b/tsDMARD treatments themselves can change the immunology of the disease. Targeting one cytokine pathway may both directly and indirectly modulate the activity of another, eg, Th1 and Th17 signalling [134], and certain pathways may be important only in specific disease manifestations or in early vs late disease [135]. For example, IL-23 signalling at sites of high mechanical stress can trigger production of IL-17, leading to independent Th17 cell activation via positive-feedback loops [128,135]. Further evidence of treatment influencing the disease course includes the development of PsA in patients with psoriasis or flares/new-onset IBD/psoriasis in patients with PsA treated with IL-17 inhibitors [136,137]. Whether or not the treatments cause, or simply unmask subclinical disease in these patients, the fact remains that bDMARD treatments may not only suppress inflammation but also manipulate it, with potentially undesirable results in some patients.

Unlike PICO1 and 3, the available literature for PICO2 is of insufficient quantity and quality to enable one to draw definitive conclusions regarding the long-term outcomes of D2M/TR PsA. The limited data that were retrieved for this SLR, however, suggest that this subset of patients with PsA experiences adverse outcomes in terms of functional and health status (as assessed by HAQ, SF-36, and EQ-5D-5L questionnaires, respectively) as well as loss of work productivity and absenteeism.

**Figure 2:**
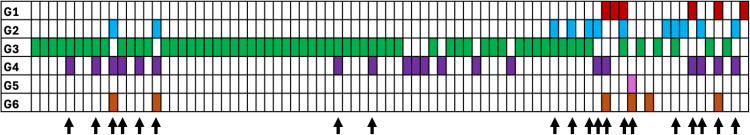
Conceptual definitions of D2M/D2T/TR PsA across studies in the systematic literature review. The figure summarises the definitions different authors use for D2M/TR PsA in all the studies included in the systematic literature review (*n* = 83 different studies; with some included in multiple PICOs). Authors’ definitions of D2T/TS PsA could be broadly divided into 6 conceptual groups (G). These were G1, red = lack/loss of response (LOR) to 1st b/tsDMARD; G2, blue = LOR to ⩾2 b/tsDMARDs, ⩾2 mechanisms of action (MOA); G3, green = LOR to b/tsDMARDs, any line of treatment/ MOA unspecified; G4, purple = failure to achieve MDA by any composite disease measure; G5, pink = objective inflammation (SJC ⩾3 OR raised CRP/ESR); G6, orange = patient/physician perception. Black arrows = studies that use multiple elements of G1-G6 to formulate their definition of D2M/TR PsA (*n* = 20). Some authors also used csDMARDs as part of the definition of D2M/TR PsA (data is not shown here). Black arrows indicate where authors of individual studies use ⩾2 elements from different conceptual groups for their definition of D2M/TR PsA. b/tsDMARDs, biologic/targeted synthetic disease-modifying antirheumatic drugs; D2M, Difficult-to-Manage; D2T, difficult-to-treat; PICO, Patients, Intervention, Comparator or Control, Outcome, Type of study; PsA, Psoriatic arthritis; TR, Treatment-Refractory.

We acknowledge that this SLR is not without limitations. Currently, there is no universally accepted definition for the explored concepts, leading to substantial heterogeneity among studies in the definitions and outcomes used, as summarised in Figure 2. This heterogeneity was also reflected in the different baseline characteristics of the populations studied. Due to these differences, we could not conduct direct comparisons or meta-analyses. In the future, harmonisation of the definition and recording of data relating to the prevalence, long-term outcomes, and predictors of D2T PsA is needed to facilitate comparisons across published cohorts and to enable more efficient and streamlined use of available data—ultimately supporting progress in research, improving real-world patient outcomes, and deepening our understanding of the health economic implications of the D2T/TR concept at local, national, and international levels. In particular, SLR data on the health economic consequences of D2T/TR PsA were scarce; however, these impacts are likely to be considerable, encompassing increased costs associated with frequent treatment switches, reduced work productivity due to persistent disease activity, and greater strain on health care systems through prolonged clinical engagement and resource utilisation.

On the other hand, a key strength of this SLR is the comprehensive and systematic screening of the available literature without predefined rules for what constitutes D2M/TR disease. We adopted an inclusive approach, including what was implied by authors of the primary studies as ‘D2T/D2M/TR’ and its individual components, even prior to the recognition or definition of these concepts. This approach contrasts with a scoping review conducted by GRAPPA, which included only articles defining this subset of patients within the last 10 years and excluded studies that failed to define D2T PsA [15]. These methodological differences explain the variation in the number of articles included and analysed. Additionally, the contribution of factors such as comorbidities and EMMs was not examined in the previous scoping review, although these are considered relevant, as reported by a recent international survey of healthcare professionals who manage patients with PsA [14].

In conclusion, this SLR has provided valuable insights into the prevalence, predictive factors, and long-term outcomes of D2M/TR PsA. Our inclusive methodology enabled a broad conceptual capture, enhancing the understanding of this patient subset. The findings underscore the importance of considering individual patient characteristics, such as comorbidities, in the management of PsA. The adverse outcomes associated with D2M/TR PsA, including functional impairment and reduced quality of life, further emphasize the need for targeted interventions. This review supports the formulation of EULAR Points to Consider for defining D2M and TR PsA.

## Editor disclosure

The peer review process did not involve Editorial Board Member Pedro Machado, and the editorial decision-making was led by editors who were not involved in the creation of this manuscript.

## Competing interests

HM-O is supported by the National Institute for Health Research (NIHR) Leeds Biomedical Research Centre (BRC) and an NIHR Senior Clinical and Practitioner Research Award. HM-O has received grant support from Janssen, Novartis, Pfizer, and UCB. Honoraria and/or speaker fees from AbbVie, Amgen, Biogen, Eli Lilly, Janssen, Moonlake, Novartis, Pfizer, Takeda, and UCB. AS has received speaking and/or consulting fees from AbbVie, Novartis, UCB, and Lilly. PMM is supported by the NIHR University College London Hospitals (UCLH) Biomedical Research Centre. PMM has received consulting/speaker’s fees from AbbVie, BMS, Celgene, Eli Lilly, Galapagos, Janssen, MSD, Novartis, Orphazyme, Pfizer, Roche, and UCB, all unrelated to this work. SRH has received speaker fees from Janssen and Novartis and sponsorship for conference attendance from Janssen, Lilly, Novartis, and UCB. GEF has received speaking and/or consulting fees from AbbVie, Novartis, UCB, Lilly, Farran, Pfizer, Janssen, MSD, GSK, AMGEN, and Boehringer Ingelheim. XM has received speaking fees and honoraria as an advisor from AbbVie, Janssen, Lilly, Novartis, and UCB. CMV has received grant support from UCB, and consultancy honoraria and/or speaker fees from AbbVie, Amgen, Janssen, Novartis, Pfizer, UCB, and Lilly. SS has received institutional research support from Amgen (previously Celgene), Boehringer Ingelheim, Bristol Myers Squibb, Eli Lilly, GSK, Janssen, and UCB and consultancy honoraria and/or speaker fees from AbbVie, Amgen, AstraZeneca, Janssen, Novartis, Pfizer, Syncona, Teijin Pharma, and UCB. LF declares no conflict of interest.

## Appendix Supplementary materials

Image, application 1

## Footnotes

Supplementary material associated with this article can be found in the online version at doi:10.1016/j.ero.2025.07.007.
