# Supplementary material for: Prevalence, outcomes, and predictive factors: a systematic literature review to inform the development of EULAR Points to Consider for the definition of Difficult-to-Manage and Treatment-Refractory psoriatic arthritis
Source: EULAR Rheumatol Open. 2025 Oct 30;2(2):100043. doi: 10.1016/j.ero.2025.07.007 (PMC13425173; doi:10.1016/j.ero.2025.07.007)
Supplement: Supplementary file 1 [file mmc1.docx]

**Online supplementary material**

Systematic literature review informing the EULAR points to consider for difficult to treat psoriatic arthritis

**Table of contents**

[**Supplementary Table 1**. Literature search strategy including MeSH terms for each database 4](#_Toc196830197)

[**Supplementary Table 2.** Baseline study characteristics for PICO1 studies 7](#_Toc196830198)

[**Supplementary Table 3.** Baseline demographic and clinical characteristics for PICO1 studies 14](#_Toc196830199)

[**Supplementary Table 4**: Real-world studies reporting D2T PsA using a definition combining failure in two or more b/tsDMARDs with persistent/high disease activity for PICO1 19](#_Toc196830200)

[**Supplementary Table 5**. Real-world studies reporting the prevalence of treatment discontinuation/switching for PICO1 1](#_Toc196830201)

[**Supplementary Table 6**. Summary of real-world studies reporting the prevalence of treatment discontinuation/switching 6](#_Toc196830202)

[**Supplementary Table 7.** Studies reporting the percentage of patients having persistent or high disease activity despite treatment with b/ts-DMARDs 7](#_Toc196830203)

[**Supplementary Table 8**: Summary of real-world studies reporting the percentage of patients having persistent or high disease activity despite treatment with b/ts-DMARDs 10](#_Toc196830204)

[**Supplementary table 9:** Long term outcomes affected by the occurrence of difficult to treat PsA 11](#_Toc196830205)

[**Supplementary Table 10**. Study characteristics for all studies included in PICO3. 1](#_Toc196830206)

[**Supplementary Table 11**. Population baseline characteristics of studies included in PICO3. 5](#_Toc196830207)

[**Supplementary Table 12.** Summary of all predictors of D2T PsA tested in PICO3 studies, including the number of statistical tests for each predictor and the percentage of positive associations with D2T PsA 7](#_Toc196830208)

[**Supplementary Table 13.** Summary of odds/risk/hazard ratio and confidence interval/p values for each study that explored sex as a predictor of D2T PsA 11](#_Toc196830209)

[**Supplementary Table 14**. Summary of odds/risk/hazard ratio and confidence interval/p values for each study that explored Age as a predictor of D2T PsA 13](#_Toc196830210)

[**Supplementary Table 15.** Summary of odds/risk/hazard ratio and confidence interval/p values for each study that explored BMI as a predictor of D2T PsA 15](#_Toc196830211)

[**Supplementary Table 16.** Summary of odds/risk/hazard ratio and confidence interval/p values for each study that explored csDMARD use as a predictor of D2T PsA 17](#_Toc196830212)

[**Supplementary Table 17.** Summary of odds/risk/hazard ratio and confidence interval/p values for each study that explored skin psoriasis as a predictor of D2T PsA 19](#_Toc196830213)

[**Supplementary Table 18**. Summary of odds/risk/hazard ratio and confidence interval/p values for each study that explored dactylitis as a predictor of D2T PsA 21](#_Toc196830214)

[**Supplementary Table 19.** Summary of odds/risk/hazard ratio and confidence interval/p values for each study that explored swollen joint count as a predictor of D2T PsA 23](#_Toc196830215)

[**Supplementary Table 20.** Summary of odds/risk/hazard ratio and confidence interval/p values for each study that explored PsA duration as a predictor of D2T PsA 25](#_Toc196830216)

[**Supplementary Table 21**. Summary of odds/risk/hazard ratio and confidence interval/p values for each study that explored tender joint count as a predictor of D2T PsA 27](#_Toc196830217)

[**Supplementary Table 22.** Summary of odds/risk/hazard ratio and confidence interval/p values for each study that explored enthesitis as a predictor of D2T PsA 29](#_Toc196830218)

#

#

# **Supplementary Table 1**. Literature search strategy including MeSH terms for each database

| **Database** | **Dates searched** | **Number of references retrieved** | **Number after de-duplication** |
| --- | --- | --- | --- |
| **MEDLINE (Ovid)**  and  Ovid MEDLINE(R) In-Process & Other Non-Indexed Citations | 1946 to March 20, 2024 | 3,843 | 3,839 |
| **Cochrane Database of Systematic Reviews**  *(Part of the Cochrane Library)* | Searched 21/03/2024 | 5 | 2 |
| **CENTRAL**  *(Part of the Cochrane Library)* | Searched 21/03/2023 | 1,477 | 1,043 |
| **EMBASE (Ovid)** | 1974 to Week 11, 2024 | 3,431 | 1,377 |
| **Epistemonikos** | Searched March 21^st^, 2024 | 279 | 172 |
| **All databases** |  | **9,038** | **6,433** |

**Medline**

1  psoriatic arthritis/
2  (psoria* adj (arthriti* or arthropath*)).tw.
3  ((arthriti* or arthropath*) adj psoria*).tw.
4  oligoarthriti*.tw.
5  (polyarthrit* or monoarthrit*).tw.
6  axial disease.tw.
7  distal interphalangeal.tw.
8  mutilans.tw.
9  or/1-8
10  Retreatment/
11  exp Treatment Failure/
12  exp "Severity of Illness Index"/
13  resistan*.tw.
14  Persisten*.tw.
15  Refractor*.tw.
16  (difficult adj2 (treat or manag*)).tw.
17  D2T.tw.
18  Inefficacy.tw.
19  (Inadequa* adj respon*).tw.
20  Intoleran*.tw.
21  failure.tw.
22  Discontinu*.tw.
23  or/10-22
24  9 and 23
25  limit 24 to (English language and humans

**Embase**

1  psoriatic arthritis/
2  (psoria* adj (arthriti* or arthropath*)).tw.
3  ((arthriti* or arthropath*) adj psoria*).tw.
4  oligoarthriti*.tw.
5  (polyarthrit* or monoarthrit*).tw.
6  axial disease.tw.
7  distal interphalangeal.tw.
8  mutilans.tw.
9  or/1-8
10  retreatment/
11  exp treatment failure/
12  "severity of illness index"/
13  resistan*.tw.
14  Persisten*.tw.
15  Refractor*.tw.
16  (difficult adj2 (treat or manag*)).tw.
17  D2T.tw.
18  Inefficacy.tw.
19  (Inadequa* adj respon*).tw.
20  Intoleran*.tw.
21  failure.tw.
22  Discontinu*.tw.
23  ("non-response" or "non response" or nonresponse or "non-responder*" or "non responder" or nonresponder*).tw.
24  or/10-23
25  9 and 24
26  limit 25 to (human and english language and (article or article in press or "review"))

**The Cochrane Library (Cochrane Database of Systematic Reviews and CENTRAL)**

#1 MeSH descriptor: [Arthritis, Psoriatic] this term only

#2 (psoria* NEXT (arthriti* or arthropath*)):ti,ab

#3 ((arthriti* or arthropath*) NEXT psoria*):ti,ab

#4 oligoarthriti*:ti,ab

#5 (polyarthrit* or monoarthrit*):ti,ab

#6 "axial disease":ti,ab

#7 "distal interphalangeal":ti,ab

#8 mutilans:ti,ab

#9 #1 OR #2 OR #3 OR #4 OR #5 OR #6 OR #7 OR #8

#10 MeSH descriptor: [Retreatment] explode all trees

#11 MeSH descriptor: [Treatment Failure] explode all trees

#12 MeSH descriptor: [Severity of Illness Index] explode all trees

#13 resistan*:ti,ab

#14 Persisten*:ti,ab

#15 Refractor*:ti,ab

#16 ((difficult NEAR/2 (treat or manag*)):ti,ab

#17 D2T:ti,ab

#18 Inefficacy:ti,ab

#19 (Inadequa* NEXT respon*):ti,ab

#20 Intoleran*:ti,ab

#21 failure:ti,ab

#22 Discontinu*:ti,ab

#23 ("non-response" or "non response" or non-responder* OR "non responders" OR non-responders OR nonresponder*):ti,ab

#24 #10 OR #11 OR #12 OR #13 OR #14 OR #15 OR #16 OR #17 OR #18 OR #19 OR #20 OR #21 OR #22 OR #23

#25 #9 AND #24

**Epistemonikos**

#1 (title:((title:(psoria*) OR abstract:(psoria*)) AND (title:(arthriti* OR arthropath*) OR abstract:(arthriti* OR arthropath*))) OR abstract:((title:(psoria*) OR abstract:(psoria*)) AND (title:(arthriti* OR arthropath*) OR abstract:(arthriti* OR arthropath*)))) OR (title:(oligoarthriti*) OR abstract:(oligoarthriti*))

#2 (title:((title:(resistan* OR Persisten* OR Refractor*.OR difficult* OR D2T OR Inefficacy. OR Inadequa* OR Intoleran* OR failure OR discontinu* OR respon*) OR abstract:(resistan* OR Persisten* OR Refractor*OR difficult* OR D2T OR Inefficacy OR Inadequa* OR Intoleran* OR failure OR discontinu* OR respon*))

#3 #1 AND #2 Limited to publication type Systematic Review

# **Supplementary Table 2.** Baseline study characteristics for PICO1 studies

| **First author and year** | **Study design** | **Definition of D2M/TR** | **No cases** | **No controls** | **RoB** |
| --- | --- | --- | --- | --- | --- |
| Zhang 2014 | Retrospective cohort | Treatment discontinuation | NA | NA | Low |
| Aaltonen 2017 | Prospective cohort | Treatment discontinuation | 3 months: 45,  6 months: 93, 12 months: 170 | 3 months: 945,  6 months: 897,  12 months: 820 | Low |
| Iannone 2017 | Prospective cohort | Treatment discontinuation | NA | NA | Low |
| Ribeiro da Silva 2019 | Retrospective cohort | Treatment discontinuation | NA | 6 months (ADA: 4571, ETN: 2920, IFX: 1105)  12months (ADA: 3387, ETN: 2142, IFX: 690)  18months (ADA: 2261, ETN: 1446, IFX: 438) | Low |
| Iannone 2019 (d1) | Retrospective cohort | Treatment discontinuation | NA | NA | Low |
| Iannone 2019 (d2) | Retrospective cohort | Not achieving DAPSA LDA/remission | NA | NA | Low |
| Michelsen 2020 | Retrospective cohort | Treatment discontinuation | NA | NA | Low |
| Hadad 2021 | Retrospective cohort | Treatment discontinuation | NA | NA | Low |
| Lindstrom 2021 (d1) | Prospective cohort | Treatment discontinuation | NA | NA | Low |
| Lindstrom 2021 (d2) | Prospective cohort | Not achieving DAPSA remission/LDA | NA | NA | Low |
| Walsh 2021 | Retrospective cohort | Treatment discontinuation | IL-12/23: 127  TNF: 176  tsDMARD: 171  IL-12/23a: 100  IL-17: 110 | NA | Low |
| Iannone 2021 (d1) | Retrospective cohort | Treatment discontinuation | NA | NA | Low |
| Iannone 2021 (d2) | Retrospective cohort | Not achieving CDAI-LDA | NA | NA | Low |
| Perrotta 2022 | Retrospective cohort | Patients must fulfil criteria A/B/C below:   1. Failure of ≥2 b/tsDMARDs with different MOAs after failing csDMARDs (unless contraindicated) 2. Signs of active disease/progressive disease, including ≥1 of: (i) moderate disease activity or worse according to validated measures, signs and symptoms of active disease, (ii) rapid radiographic progression, (iii) or PsA symptoms leading to reduced QoL 3. PsA deemed problematic by rheumatologist and/or patient | 36 | 70 | Low |
| Gossec 2022 (d1) | Prospective observational | Treatment discontinuation | UST: 317, TNFi: 321 | NA | Low |
| Gossec 2022 (d2) | Prospective observational | Not achieving MDA/DAPSA LDA/remission | UST: 317, TNFi: 321 | NA | Low |
| Eviatar 2022 | Retrospective cohort | Treatment discontinuation | NA | NA | Low |
| Gossec 2023 (d1) | Prospective observational | Treatment discontinuation | UST: 217, TNF: 218 | NA | Low |
| Gossec 2023 (d2) | Prospective observational | Not achieving MDA/DAPSA LDA/remission | UST: 217, TNF: 218 | NA | Low |
| Mease 2023a | Prospective observational | Treatment discontinuation | 104 all, 90 on-label | NA | Low |
| Vassilakis 2024 (d1) | Retrospective cohort | Patients must fulfil criteria A/B below:   1. Failure of ≥2 csDMARD (unless contraindicated) and ≥2 b/ts DMARDs (except for apremilast) with different MOAs 2. Fulfill MODA definition (DAPSA) and/or were not in MDA at the time of assessment | 77 | 390 | Low |
| Vassilakis 2024 (d2) | Retrospective cohort | Patients must fulfil criteria A/B below:   1. Failure of ≥2 csDMARD (unless contraindicated) and ≥2 b/ts DMARDs (except for apremilast) with different MOAs 2. Fulfill MODA definition (DAPSA) at the time of assessment | 55 | 673 | Low |
| Vassilakis 2024 (d3) | Retrospective cohort | Patients must fulfil criteria A/B below:   1. Failure of ≥2 csDMARD (unless contraindicated) and ≥2 b/ts DMARDs (except for apremilast) with different MOAs 2. Not in MDA at the time of assessment | 49 | 344 | Low |
| Perrotta 2024 (d1) | Retrospective cohort | Patients must fulfil criteria A/B/C below:   1. Failed ≥2 csDMARD and ≥2 b/tsDMARDs with different MOAs 2. Signs of active disease/progressive disease, including ≥1 of: (i) at least moderate disease activity according to validated measures, signs and symptoms of active disease, (ii) rapid radiographic progression, or (iii) PsA symptoms leading to reduced QoL 3. Deemed problematic by rheumatologist and/or patient | 75 | 303 | Low |
| Perrotta 2024 (d2) | Retrospective cohort | Patients must fulfil criteria A/B below:   1. Failed ≥2 csDMARD and ≥2 b/tsDMARDs with different MOAs 2. Patients with ≥1 of the following: (i) moderate disease activity (DAPSA ≥14 or PASDAS ≥3.2), (ii) signs and symptoms of active MSK disease, (iii) axial PsA, (iv) active skin disease, (v) inability to taper GC <7.5mg/day, (vi) high CRP, g. rapid radiographic progression | 58 | 320 | Low |
| Coates 2008 | Retrospective cohort | Treatment discontinuation | 3 | 57 | Low |
| Oelke 2019 | Retrospective cohort | Treatment discontinuation | ADA: 307  ETN: 203  CZP: 48  GOL:29  SEC: 93 | ADA: 413  ETN: 223  CZP: 45  GOL:35  SEC: 162 | Low |
| D´Angelo 2019 | Retrospective cohort | Treatment discontinuation | 1st year: 57  2nd year: 444 | 1st year: 367  2nd year: 313 | Low |
| Rahman 2020 | Prospective cohort | Treatment discontinuation | IFX: 70  GOL: 143  UST: 34 | IFX: 41  GOL: 138  UST: 36 | Low |
| Zagni 2020 | Retrospective cohort | Treatment discontinuation | NA | NA | Low |
| Azuaga 2020 | Retrospective cohort | Treatment discontinuation | 72 | 129 | Low |
| Pina-Vegas 2022 | Retrospective cohort | Treatment discontinuation | NA | NA | Low |
| Pombo Suarez-2022 | Retrospective cohort | Treatment discontinuation | 1st year: 72  8th year: 155 | 1st year: 176  8th year: 93 | Low |
| Pina-Vegas 2023 | Retrospective cohort | Treatment discontinuation | NA | NA | Low |
| Favalli 2020 | Retrospective cohort | Treatment discontinuation | NA | NA | Low |
| Saad 2010 | Retrospective cohort | Treatment discontinuation | 177 | 419 | Mod |
| Glintborg 2011 (d1) | Prospective cohort | Treatment discontinuation | 336 | 428 | Mod |
| Glintborg 2011 (d2) | Prospective cohort | Not achieving EULAR good response | 336 | 428 | Mod |
| Soubrier 2015 | Retrospective cohort | Treatment discontinuation | NA | NA | Mod |
| Harrold 2017 | Retrospective cohort | Treatment discontinuation | 571 | 670 | Mod |
| Stober 2018 | Retrospective cohort | Treatment discontinuation | 3 months: 5  6 moths: 22  12 months: 55  24 months: 75 | 3 months: 183  6 months: 166  12 months: 133  24 months: 113 | Mod |
| Ribero Da Silva-2019a | Retrospective cohort | Treatment discontinuation | 6 months: 27  12 months: 54 | 6 months:134  12 months: 107 | Mod |
| Sewerin 2021 | Retrospective cohort | Treatment discontinuation | Total: 148  ADA:44  CZP: 14  ETN: 49  GOL: 8  IFX: <5  SEC: 24  UST: 6 | Total: NA  ADA: 61  CZP: 15  ETN: 51  GOL: 12  IFX: NA  SEC: 34  UST: 26 | Mod |
| Jin 2021 | Retrospective cohort | Treatment discontinuation | 12 months, Total: 2594  12 months, TNFi: 2495  12 months, IL-17i: 99 | 12 months, Total: NA  12 months, TNFi: 1685  12 months, IL-17i: 115 | Mod |
| Mateo Soria 2022 | Retrospective cohort | Treatment discontinuation | 96 | 63 | Mod |
| Mease 2023 | Prospective cohort | Treatment discontinuation | NA | NA | Mod |
| Joven 2023 | Retrospective cohort | Treatment discontinuation | NA | NA | Mod |
| Vadhariya 2023 (abstract) | Retrospective cohort | Treatment discontinuation | NA | NA | Mod |
| Mease 2024 | Prospective cohort | Treatment discontinuation | 63 | NA | Mod |
| Alp 2024 | Retrospective cohort | Patients must fulfil criteria A/B below:   1. Failure of ≥2 csDMARD and ≥2b/tsDMARDs with different MOAs 2. DAPSA ≥14 | 33 | 138 | Mod |
| Ferrito 2024 (abstract) | Prospective cohort | Patients must fulfil criteria A/B below:   1. Failure of csDMARDs and ≥2b/tsDMARDs with different MOAs 2. Signs/symptoms of progressive disease | 8 | 259 | Mod |
| Harrison 2024 (abstract) | Retrospective cohort | Patients must fulfil criteria A/B below:   1. Failure of ≥2 b/tsDMARDs 2. DAPSA ≥14 | 91 | 42 | Mod |
| Mazzota 2009 | Prospective cohort | Treatment discontinuation | 5 | 7 | Mod |
| Virkki 2010 | Retrospective cohort | Treatment discontinuation | 46 | 63 | Mod |
| Kristenser 2016 (d1) | Prospective observational | Treatment discontinuation | 99 | 175 | Mod |
| Kristenser 2016 (d2) | Prospective observational | Not achieving ACR20/50, EULAR good response | 99 | 175 | Mod |
| Luy 2016 | Retrospective cohort | Treatment discontinuation | 79 | 118 | Mod |
| Favalli 2017 | Retrospective cohort | Treatment discontinuation | 143 | 155 | Mod |
| Walsh 2018 | Retrospective cohort | Treatment discontinuation | 686 | Total: 549  ADA: 126  CZP: 8  ETN: 239  GOL: 52  IFX: 79  UST: 45 | Mod |
| Iannone 2018 (d1) | Retrospective cohort | Treatment discontinuation | NA | NA | Mod |
| Iannone 2018 (d2) | Retrospective cohort | Not achieving DAPSA remission | NA | NA | Mod |
| Rotar 2019 | Prospective cohort | Treatment discontinuation | 12 months, GOL: 23  24 months, GOL: 34  12 months, other TNFi: 98  2 years, other TNFi: 135 | 12 months, GOL: 60  24 months, GOL: 49  12 months, other TNFi: 230  2 years, other TNFi: 193 | Mod |
| Gonzalez Fernandez-2019 | Retrospective cohort | Treatment discontinuation | 8 | 24 | Mod |
| Murage 2021 | Retrospective cohort | Treatment discontinuation | Total: 294  Discontinued: 192 Switched: 102 | Total: 262 | Mod |
| Moreno-Ramos 2022 | Retrospective cohort | Not achieving remission (DAS28-CRP<2.6) or low disease activity (DAS28-CRP 2.6-3.2) | NA | NA | Mod |
| Ferrito 2023 | Retrospective cohort | Treatment discontinuation | TNFi: 21  nonTNFi: 39 | TNFi: 134  non-TNFi: 47 | Mod |
| Philippoteaux 2023 | Retrospective cohort | Patients who received b/tsDMARDs with different MOAs | 49 | 101 | Mod |
| Gentiloni 2023 | Retrospective cohort | Not achieving MDA, DAPSA, ASDAS-CRP inactive disease | NA | NA | Mod |
| D’Angelo 2024 | Retrospective cohort | Not achieving MDA, DAS28-CRP, EULAR good/moderate response | NA | NA | Mod |
| Lopalco 2024 (abstract) | Retrospective cohort | Not achieving MDA | NA | NA | Mod |
| Pantano 2024 (abstract) | Retrospective cohort | DAPSA remission/LDA | NA | NA | Mod |
| Saad 2009 | Retrospective cohort | Treatment discontinuation | 103 | 319 | Mod |
| Galindez Agirregoiko2021a | Prospective cohort | Treatment discontinuation | 20 | 57 | Mod |
| Ruscitti 2024 | Retrospective cohort | DAPSA ≥14 despite treatment | NA | NA | Mod |
| Campanilho Marques 2010 | Retrospective cohort | Treatment discontinuation | 14 | 28 | High |
| Bonafede 2012 | Retrospective cohort | Treatment discontinuation | Total: 575  Restart after >45 days gap:268  Switch:121  Discontinue: 186 | 581 | High |
| Iervolino 2012 | Prospective cohort | Treatment discontinuation | 10 | 136 | High |
| Fagerli 2013 | Prospective cohort | EULAR good response or switch at 3 months | 95 | 344 | High |
| Takami 2023 | Retrospective cohort | Treatment discontinuation | 63 | 83 | High |
| Pons 2023 (abstract) | Retrospective cohort | Treatment discontinuation | 12 months: 361  2 years: 498 | 12 months: 614  2 years: 477 | High |
| Erik 2023 (abstract) | Retrospective cohort | Treatment discontinuation | 12 months: 465  2 years: 624 | 12 months: 314  2 years:155 | High |

**Key:** ADA= adalimumab; ASDAS= axial spondyloarthritis disease activity score; CDAI= clinical disease activity index; CZP= certolizumab pegol, CRP= C-reactive protein, DAPSA= disease activity psoriatic arthritis, ETN= etanercept, GC= glucocorticoids, GOL= golimumab; GUS= guselkumab; IFX= infliximab, IL; interleukin, IL-17i: Interleukin 17 inhibitor; LDA= low disease activity; MDA= minimal disease activity; MOA= mechanisms of action; Mod = moderate; MODA= moderate disease activity; MSK= musculoskeletal; NA= not applicable; PASDAS= psoriatic arthritis disease activity score, PsA= psoriatic arthritis; QoL= quality of life; RIS= risankizumab; SEC= secukinumab; TNF= tumor necrosis factor; TNFi= TNF inhibitor; UPA= Upadacitinib; UST= Ustekinumab; VLDA= very low disease activity.

# **Supplementary Table 3.** Baseline demographic and clinical characteristics for PICO1 studies

| **First author (year)** | | **Male sex (n/ %)** | **BMI (mean/SD)** | **PsA disease duration in years**  **(mean/SD or range)** | **Age at study entry in years**  **(mean/SD, years)** | **PsO ever**  **(n/ %)** | **IBD ever**  **(n/ %)** | **Uveitis ever**  **(n/ %)** | **Axial involvement ever**  **(n/ %)** |
| --- | --- | --- | --- | --- | --- | --- | --- | --- | --- |
| Zhang 2014 | | 1,512/ 46.3 | NA | NA | 49.4/ 11.6 | NA | NA | NA | NA |
| Aaltonen 2017 | | NA | NA | NA | NA | NA | NA | NA | NA |
| Iannone 2017 | | 128/ 70.0 | 27.5/5.0 | 6.9/6.0 | 53/10 | NA | NA | NA | NA |
| Ribeiro da Silva 2019 | | 5,679/ 51.6 | NA | NA | 47.7/  13.1 | NA | NA | NA | NA |
| Iannone 2019 | | 68/ 72 | 29.1/5 | 8.7/8 | 44.8/9 | NA | NA | NA | NA |
| Michelsen 2020 | | NA/ 55.0 | NA | 8.6/9.0 | 48.1/11.7 | NA | NA | NA | NA |
| Hadad 2021 | | 107/ 55 | NA | NA | 46.8/11.5 | NA | NA | NA | NA |
| Lindstrom 2021 | | NA | NA | NA | NA | NA | NA | NA | NA |
| Walsh 2021 | | NA | NA | NA | NA | NA | NA | NA | NA |
| Iannone 2021 | | 138/ 54 | NA | 6.1/ 6.0 | 51/ 12 | NA | 2.0/ 0.8 | 3.0/ 1.2 | NA |
| Gossec 2023 | UST | 246/ 56.2 | 28.6/ 6.2 | 7.5/ 8.1 | 51.0/ 12.5 | NA | NA | NA | NA |
|  | TNFi | 248/ 54.5 | 27.7/ 5.3 | 6.2/ 6.6 | 48.5/ 12.5 | NA | NA | NA | NA |
| Eviatar 2022 | | NA | NA | NA | NA | NA | NA | NA | NA |
| Gossec 2023 | UST | 192/ 43.7 | 28.6/ 6.2 | 7.5/ 8.1 | 51.1/ 12.5 | NA | NA | NA | NA |
|  | TNFi | 208/ 45.6 | 27.8/ 5.3 | 6.2/ 6.6 | 48.5/ 12.6 | NA | NA | NA | NA |
| Mease 2023a | | NA | NA | NA | NA | NA | NA | NA | NA |
| Coates 2008 | | 33/ 55 | NA | 16/ 2-41 | 46 | NA | NA | NA | NA |
| Oelke 2019 | | NA | NA | NA | NA | NA | NA | NA | NA |
| D´Angelo 2019 | | 227/ 53.5 | 25.8/ 4.4 | 7.6/ 7.2 | 53.7/ 11.3 | NA | 35.0/ 8.2 | 27/ 6.4 | 81.0/ 19.1 |
| Rahman 2020 | | NA | NA | NA | NA | NA | NA | NA | NA |
| Zagni 2020 | | NA | NA | NA | NA | NA | NA | NA | NA |
| Azuaga 2020 | | NA | 27.6/ 30.0 | NA | 53/ NA | NA | NA | NA | 2/1 |
| Pina-Vegas 2022 | | 1028.0/ 34.5 | NA | NA | 47.4/ 12.4 | NA | 235.0/ 7.9 | 15.0/ 0.5 | NA |
| Pombo Suarez 2022 | | 110.0 /44.4 | NA | 7.1/  3.0-12.5 | 50.2/ 11.6 | NA | NA | NA | NA |
| Pina-Vegas 2023 | | NA/ 43 | NA | NA | 50.5/ 13.1 | NA | 788/ 8.3 | 44/ 0.3 | NA |
| Favalli 2020 | | 65.0/ 63.7 | 25.6/  4.79 | 7.1/5.1 | 51.7/ 10.6 | NA | NA | NA | NA |
| Glintborg 2011 | | 368/ 48 | NA | 5/2-11 | 47/ 38-56 | NA | NA | NA | NA |
| Soubrier 2015 | | 107/ 55 | NA | NA | 46.8/ 11.5 | NA | NA | NA | NA |
| Harrold 2017 | | 566.0/ 45.3 | NA | 8.3/ 8.5 | 50.7/ 12.1 | NA | NA | NA | NA |
| Stober 2018 | | 92/ 49 | 32.6/ 7.4 | 11/ 7-16 | 47.2/ 11.4 | NA | NA | NA | NA |
| Ribero Da Silva 2019 | | 70.0/ 43.5 | 26.4/  23.9-29.9 | NA | 51.6/  42.5-59.5 | 139.0/  95.9 | NA | NA | 83.0/ 60.6 |
| Sewerin 2021 | | 50.5/ 11.7 | NA | NA | 50.4/ 12.4 | NA | NA | NA | NA |
| Perrotta 2022 | | NA | NA | NA | NA | NA | NA | NA | NA |
| Vassilakis 2024 | | NA | NA | NA | NA | NA | NA | NA | NA |
| Perrotta 2024 | | NA | NA | NA | NA | NA | NA | NA | NA |
| Alp 2024 | | 116.0/ 67.8 | 29.2/ 5.0 | NA | 48.2/  11.2 | NA | NA | NA | 22.0/ 12.9 |
| Philippoteaux 2023 | | 29.0/ 59.2 | 29.6/ 7.2 | 17/ 8-20 | 54.3/ 11.9 | NA | 0 | 1/ 2 | 21/43.8 |
| Ferrito 2024 (abstract) | | 140/52.4 | NA | NA | 52/46-61^ | NA | NA | NA | 29/10.9 |
| Harrison 2024 (abstract) | | 61.0/ 45.9 | 32.0/ 7.5 | 13.0/ 7.9 | 52.3/ 12.8 | NA | 10/7.5 | 13.0/ 9.8 | NA |
| Jin 2021 | | 43 | NA | NA | 50.4/ 11.7 | 63.2 | NA | NA | NA |
| Mateo Soria 2022 | | 79/ 49.7 | NA | NA | NA | 131.0/  85.6 | NA | NA | 9/ 6 |
| Mease 2023 | | 571/ 57 | 32.8/ 7.9 | 6.7/ 7.9 | 52.9/ 12.3 | NA | NA | NA | 199/ 20 |
| Joven 2023 | | 114/ 51.6 | 28.6/ 6.3 | NA | 51.5/ 11.6 | NA | NA | NA | NA |
| Mease 2024 | | NA | NA | NA | NA | NA | NA | NA | NA |
| Mazzota 2009 | | 21.0/ 65.6 | NA | 11.2/ 7.7 | 53.0/ 11.0 | NA | NA | NA | NA |
| Kristenser 2016 | 1^st^ time switchers | 42/ 91 | NA | 7.3/ 3.9-13.8 | 47/ 38-56 | NA | NA | NA | NA |
|  | 2^nd^ time switchers | 40/ 23 | NA | 9.3/ 5.5-15.3 | 48/ 38-56 | NA | NA | NA | NA |
| Luy 2016 | | 98.0/ 49.7 | NA | NA | 49.4/ 10.6 | 1.0/ 0.5 | NA | NA | NA |
| Favalli 2017 | | 154.0/ 51.7 | NA | 8.8/ 7.7 | 47.8/ 12.1 | NA | NA | NA | NA |
| Rotar 2019 | | 41/ 49 | NA | 8.8/ 4.0-15.5 | 51.0/ 44.4-56.6 | NA | NA | NA | NA |
| Gonzalez Fernandez 2019 | | 22.0/ 66.6 | NA | NA | 48.82/ 11.2 | NA | NA | NA | NA |
| Murage 2021 | | 246.0/ 49.6 | NA | NA | 51.1/ 9.8 | 463.0/ 93.4 | 7.0/ 1.4 | 6.0/ 1.2 | NA |
| Ferrito 2023 | | NA | NA | NA | NA | NA | NA | NA | NA |
| Saad 2009 | | NA | NA | NA | NA | NA | NA | NA | NA |
| Galindez Agirregoikoa 2021 | | 59.0/ 67.8 | NA | 12.3/ 9.3 | 52.8/ 11.4 | NA | NA | NA | NA |
| Takami 2023 | | NA | NA | NA | NA | NA | NA | NA | NA |
| Bonafede 2012 | | NA | NA | NA | NA | NA | NA | NA | NA |
| Campanilho Marques 2010 | | 23/ 55 | NA | 10.7/ 5.6 | 49.8/ 10.9 | NA | NA | NA | NA |
| Vadhariya 2023 (abstract) | | NA | NA | NA | NA | NA | NA | NA | NA |
| Pons 2023 (abstract) | | 429/ 44 | NA | NA | 52/ 44-59 | NA | NA | NA | NA |
| Erik 2023 (abstract) | | NA | NA | NA | NA | NA | NA | NA | NA |
| Saad 2010 | | 280/ 47 | NA | 12.4/ 8.7 | 45.7/ 11.1 | NA | NA | NA | NA |
| Virkki 2010 | | 75.0/ 59.1 | NA | 11/ 0-35 | 50/ 20-73 | NA | NA | NA | NA |
| Walsh 2018 | | 587.0/ 47.5 | NA | NA | 50.3/ 12.1 | NA | NA | NA | NA |
| Iannone 2018 | | 84/ 52 | 28.2/ 5.0 | 9.5/ 6.5 | 53.6/ 10.0 | NA | NA | NA | 8/ 5 |
| Iervolino 2012 | | 58.0/ 42.6 | NA | 5.19/3.04 | 45.62/11.82 | NA | NA | NA | 46/33.8 |
| Fagerli 2013 | | NA | NA | NA | NA | NA | NA | NA | NA |
| D’Angelo 2024 | | 47.0/ 51.6 | 27.2/ 5.0 | 9.8/ 7.8 | 53.7/ 11.3 | NA | NA | NA | NA |
| Moreno-Ramos 2022 | | 403.0/ 55.7 | NA | 7.0/ 2.7–14.4 | 51.6/ 11.9 | NA | NA | NA | NA |
| Gentiloni 2023 | | 40.0/ 31.7 | 26.7/ 5.1 | 92/ 45–177 | 56.5/ 11.4 | NA | 6.0/ 4.8 | 6.0/ 4.8 | 54.0/ 42.9 |
| Ruscitti 2024 | | NA/ 20.7 | 25.5/ 5.1 | 6.0/ 7.0 | 56.8/ 9.9 | NA | NA | NA | NA/ 64.3 |
| Lopalco 2024 abs | | 72.0/ 28.5 | NA | 111.2/ 102.5 | 55.8/ 10.9 | NA | NA | NA | 92.0/ 36.4 |
| Pantano 2024 (abstract) | | 28.0/ 63.6 | 29.1 | 11.4 | 56.2 | NA | NA | NA | NA |

**Key:** % = percentage; BMI = body mass index kg/m^2^; IBD = inflammatory bowel disease; n= number; NA = not applicable; PsA = psoriatic arthritis; PsO = psoriasis; SD = standard deviation.

# **Supplementary Table 4**: Real-world studies reporting D2M/TR PsA using a definition combining failure in two or more b/tsDMARDs with persistent/high disease activity for PICO1

| **First author and year** | **Study design** | **Definition of D2M/TR** | | **Meet criteria for D2M/TR (%)*** |
| --- | --- | --- | --- | --- |
|  |  | **number of b/ts DMARDs received** | **Disease activity** |  |
| Perrotta 2022 | Retrospective cohort | Patients fulfil A (below) and criteria B/C (right)   1. Failure of ≥2 b/tsDMARDs with different MOAs after failing csDMARDs (unless contraindicated) | 1. Signs of active disease/progressive disease, including ≥1 of: (i) moderate disease activity or worse according to validated measures, signs and symptoms of active disease, (ii) rapid radiographic progression, (iii) or PsA symptoms leading to reduced QoL 2. PsA deemed problematic by rheumatologist and/or patient | 33.6 |
| Vassilakis 2024 (d1) | Retrospective cohort | Patients fulfil A (below) and B (left):   1. Failure of ≥2 csDMARD (unless contraindicated) and ≥2 b/ts DMARDs (except for apremilast) with different MOAs | 1. Fulfill MODA definition (DAPSA) and/or were not in MDA at the time of assessment | 16.5 |
| Vassilakis 2024 (d2) | Retrospective cohort | Patients fulfil A (below) and B (left):   1. Failure of ≥2 csDMARD (unless contraindicated) and ≥2 b/ts DMARDs (except for apremilast) with different MOAs | 1. Fulfill MODA definition (DAPSA) | 7.6 |
| Vassilakis 2024 (d3) | Retrospective cohort | Patients fulfil A (below) and B (left):   1. Failure of ≥2 csDMARD (unless contraindicated) and ≥2 b/ts DMARDs (except for apremilast) with different MOAs | 1. Not in MDA at the time of assessment | 12.5 |
| Perrotta 2024 (d1) | Retrospective cohort | Patients fulfil A (below) and B/C (left):   1. Failed ≥2 csDMARD and ≥2 b/tsDMARDs with different MOAs | 1. Signs of active disease/progressive disease, including ≥1 of: (i) at least moderate disease activity according to validated measures, signs and symptoms of active disease, (ii) rapid radiographic progression, or (iii) PsA symptoms leading to reduced QoL 2. Deemed problematic by rheumatologist and/or patient | 19.8 |
| Perrotta 2024 (d2) | Retrospective cohort | Patients fulfil A (below) and B (left):   1. Failed ≥2 csDMARD and ≥2 b/tsDMARDs with different MOAs | 1. Patients with ≥1 of the following: (i) moderate disease activity (DAPSA ≥14 or PASDAS ≥3.2), (ii) signs and symptoms of active MSK disease, (iii) axial PsA, (iv) active skin disease, (v) inability to taper GC <7.5mg/day, (vi) high CRP, g. rapid radiographic progression | 15.3 |
| Alp 2024 | Retrospective cohort | Failure of ≥1 csDMARD and ≥2 b/tsDMARDs with different MOA | DAPSA ≥14 | 19.3 |
| Philippoteaux 2023 | Retrospective cohort | Failure of ≥2 b/tsDMARDs with different MOA | NA | 32.7 |
| Ferrito 2024 (abstract) | Prospective cohort | Failure of csDMARDs and ≥2 b/tsDMARDs with different MOA | Signs/symptoms of progressive disease | 2.9 |
| Harrison 2024 (abstract) | Retrospective cohort | Failure of ≥2 b/tsDMARDs | DAPSA ≥14 | 68.4 |

**Key**: DAPSA= disease activity psoriatic arthritis; D2T= difficult to treat; DMARD= disease-modifying antirheumatic drug; GC= glucocorticoid; MOA= mechanism of action; MODA= moderate disease activity; MSK= musculoskeletal; MDA= minimal disease activity; PASDAS= psoriatic arthritis disease activity score; PsA=psoriatic arthritis; QoL= quality of life. *All percentages are reported to 1 decimal place.

# **Supplementary Table 5**. Real-world studies reporting the prevalence of treatment discontinuation/switching in D2M/TR PsA for PICO1

| **First author and year** | **Type of study** | **Treatment** | **Prevalence of treatment discontinuation (%)*** | |
| --- | --- | --- | --- | --- |
|  |  |  | **Time point** | **%** |
| Coates 2008 | Retrospective cohort | Treated with INF, ETN, ADA | 3 months | 5 |
| Zhang 2014 | Retrospective cohort | Starting bDMARD | 1 month | 8 |
|  |  |  | 3 months | 20 |
|  |  |  | 6 months | 33 |
|  |  |  | 12months | 46 |
| Aaltonen 2017 | Prospective cohort | Starting TNFi | 3months | 4.5 |
|  |  |  | 6months | 9.4 |
|  |  |  | 12months | 17 |
| Iannone 2017 | Prospective cohort | Starting GOL | 24 months | Total: 33.1, male: 26.4, female: 35.9 |
| Ribeiro da Silva 2019 | Retrospective cohort | PsA treated with TNFi (ADA, ETN, IFX) | 6 months | ADA: 20, ETN: 22.6, IFX: 27.5 |
|  |  |  | 12 months | ADA: 40.7, ETN: 43.3, IFX: 54.7 |
|  |  |  | 18 months | ADA: 52.9, ETN: 55.2, IFX: 67.0 |
|  |  |  | 24 months | ADA: 59.6, ETN: 61.1, IFX: 75.6 |
| Iannone 2019 | Retrospective cohort | PsA, starting CZB | mean survival: 25 months | 37.2 |
| Oelke 2019 | Retrospective cohort | PsA with $\geq1$ insurance claim for ADA, CZP, ETN, GOL, SEC | 12 months | ADA: 42.6, CZP: 51.6, ETN: 47.7, GOL: 45.3, SEC: 36.5 |
| D´Angelo 2019 | Retrospective cohort | PsA initiating ADA as 1st or 2nd line | 12months | 13.4 |
|  |  |  | 24months | 27.2 |
| Michelsen 2020 | Retrospective cohort | Starting GOL | 12 months | bio-exp, bio-naïve: 36, 43 |
|  |  |  | 24 months | 71.2 |
|  |  |  | 48 months | 87.3 |
| Rahman 2020 | Prospective cohort | PsA initiating IFX, GOL or UST | mean exposure: 2.9y | IFX: 63.1 |
|  |  |  | mean exposure: 1.9y | GOL: 50.9 |
|  |  |  | mean exposure: 1.2y | UST: 48.6 |
| Zagni 2020 | Retrospective cohort | Receiving bDMARDs on hospital discharge | 6 months | ADA: 22, CMB: 19, ETN: 23, GOL: 22, IFX: 29, SEC: 15, UST: 15 |
| Azuaga 2020 | Retrospective cohort | PsA initiating UST | average 17.7 months | 35.9 |
| Hadad 2021 | Retrospective cohort | PsA with $\geq$2 consecutive dispensed prescriptions with any bDMARD | 12 months | ADA: 47.3, ETN:43.6, IFX: 48.6, GOL: 45, UST: 55.4, SEC:36.8 |
|  |  |  | 24 months | ADA: 63, ETN:58.8, IFX: 66, GOL: 59.5 UST: 68.1, SEC: 58.3 |
|  |  |  | 60 months | ADA: 78.7, ETN: 76.7, IFX: 82.5, GOL: 77.5, UST: 82.7 |
| Lindstrom 2021 | Prospective cohort | PsA starting SEC, ADA, CZP, ETN, GOL, IFX | 12 months | 1^st^ line: ADA: 30, SEC: 30, ETA: 42, IFX: 34, GOL: 36, CZP: 36  2^nd^ line: ADA: 36, SEC: 34, ETA: 39, IFX: 38, GOL:34, CZP: 52  3^rd^ line: ADA: 33, SEC: 40, ETA: 40, IFX: 46, GOL 46, CZP: 57 |
| Walsh 2021 | Retrospective cohort | PsA initiating b/tsDMARDs | 12 months | IL-12/23i: 53.4, TNFi: 73.9, tsDMARDs: 71.8, IL-12/23i: 52.9, IL-17i: 58.2 |
| Iannone 2021 | Retrospective cohort | PsA starting GOL | 24 months | naïve: 18.2, 1 previous bDMARD: 18.2, 2 previous bDMARD: 17.5 |
| Gossec 2022 | Prospective observational | PsA treated with UST or TNFi | 12 months | UST: 27.6, TNF: 29.5 |
| Eviatar 2022 | Retrospective cohort | PsA on SEC or TNFi | 12 months | SEC: 8, ETN: 24, IFX: 19, ADA: 21, GOL:40 |
|  |  |  | 24 months | SEC: 8, ETN: 51, IFX: 37, ADA:37, GOL:57 |
|  |  |  | 36 months | SEC: 24, ETN: 50, IFX: 48, ADA: 44, GOL66 |
| Pina-Vegas 2022 | Retrospective cohort | PsA starting a second bDMARD after TNFi failure | 12 months | Overall/TNFi/IL-17i/IL-12-23i  Overall: 57.4, TNFi: 63.2, IL-17i: 53.7, IL-23/12i: 44.4 |
|  |  |  | 24 months | Overall: 75, TNFi: 78.7, IL-17i: 72, IL-23/12i: 68.1 |
|  |  |  | 36 months | Overall: 82.8, TNFi: 84.9, IL-17i: 81.0, IL-23/12i: 79.4 |
| Pombo Suarez 2022 | Retrospective cohort | Patients taking GOL | 12 months | 28.9 |
|  |  |  | 8 year | 62.3 |
| Gossec 2023 | Prospective observational | PsA treated with UST or TNFi | 12 months | UST: 16.4, TNFi: 20.0 |
|  |  |  | 24 months | UST: 38.5, TNFi: 37.9 |
|  |  |  | 36 months | UST: 50.1, TNFi: 52.2 |
| Mease 2023a | Prospective observational | PsA initiating GUS | 6 months | 21.8 |
| Pina-Vegas 2023 | Retrospective cohort | PsA starting a b/tsDMARD except apremilast | 12 months | Females/males/bDMARD-exp Females/bDMARD-naive Males  47.8/37.6/45.6/33.2 |
|  |  |  | 24 months | 64.5/52.9/61.7/47.9 |
|  |  |  | 36 months | 73.1/60.7/69.9/55.6 |
| Favalli 2020 | Retrospective cohort | PsA treated with ADA after first-line ETN failure | 24 months | 43.5 (44 with MTX, 37.8 without MTX) |
| Saad-2009 | Retrospective cohort | PsA treated with TNFi | 12 months | 24.5 |
| Mazzota 2009 | Prospective cohort | PsA treated with ETN | 12 months | 15.6 |
| Saad-2010 | Retrospective cohort | PsA initiating ETN, ADA, INF | 12 months | 29.7 |
| Virkki 2010 | Retrospective cohort | PsA initiating INF, ETN, ADA, ANK | 24 months | 36.2 |
| Glintborg 2011 | Prospective cohort | PsA, treated with TNFi | 24 months | 44 |
| Soubrier 2015 | Retrospective cohort | PsA starting 1st TNFi | 3 months | 10 |
| Kristenser 2016 | Prospective cohort | PsA initiating TNFi | 6 months | 36.1 |
| Luy 2016 | Retrospective cohort | PsA initiating subcutaneous bDMARDs | 12 months | 40.3 |
| Harrold 2017 | Retrospective cohort | PsA with or without prior bDMARDs initiating TNFi | 4 years | 46 |
| Favalli 2017 | Retrospective cohort | PsA treated with a 1st line TNFi | 8 years: | 52 |
| Iannone 2018 | Retrospective cohort | PsA starting UST | 12months: | naïve: 13, TNF-IR: 32 |
| Stober 2018 | Retrospective cohort | PsA treated with ETN or ADA | 3 months | 2.7 |
|  |  |  | 6 months | 11.7 |
|  |  |  | 12 months | 29.3 |
|  |  |  | 24 months | 39.9 |
| Walsh 2018 | Retrospective cohort | PsA initiating bDMARD | 12 months | 55.5 |
| Ribero Da Silva 2019 | Retrospective cohort | PsA treated with TNFi | 6 months | 16.7 |
|  |  |  | 12 months | 33.5 |
| Rotar 2019 | Prospective cohort | PsA treated with GOL or other TNFi | 12 months | GOL: 28, Non-TNFi: 28 |
|  |  |  | 24 months | GOL: 41, Non-TNFi: 41 |
| Gonzalez Fernandez 2019 | Retrospective cohort | PsA starting bDMARD | 8 years: | 27.3 |
| Sewerin 2021 | Retrospective cohort | PsA starting bDMARDs | 12 months | ADA: 41.9, CZP: 48.3, ETN: 49, GOL:40, IFX: <5%, SEC: 45.3, UST: 18.8% |
| Jin 2021 | Retrospective cohort | PsA patients initiating TNFi or IL inhibitors | 12 months | TNFi: 47, ILi: 46.3 |
| Murage 2021 | Retrospective cohort | PsA initiating IXE | 12 months | 59.3 |
| Mateo Soria 2022 | Retrospective cohort | PsA receiving ≥1 bDMARD or APR | 12 months | 60.0 |
| Mease 2023 | Prospective cohort | PsA starting TNFi or IL-17i | 6 months | TNFi: 32, IL-17i: 32, ETN: 37 |
| Joven 2023 | Retrospective cohort | PsA starting IL-17i | 3 months | SEC150: 1, SEC300: 8.1, IXE: 2.5 |
|  |  |  | 6 months | SEC150: 9, SEC 300: 33.3, IXE: 5.1 |
|  |  |  | 12 months | SEC 150: 17, SEC300: 35.5, IXE: 13.6 |
| Ferrito 2023 | Retrospective cohort | PsA started TNFi, anti-IL-17, IL-12/IL-23 | 24 months | TNF: 13.5, Non-TNF: 49.3 |
| Gentiloni 2023 | Retrospective cohort | PsA starting UPA | 12 months | 10 |
| Vadhariya 2023 (abstract) | Retrospective cohort | PsA on IL-17Ai or TNFi | 3 months: | IL-17i: 16.2, TNFi: 19.3 |
|  |  |  | 9 months: | IL-17i: 40.9, TNFi: 49.1 |
|  |  |  | 12 months: | IL-17: 49.1, TNFi: 57.1 |
| Mease 2024 | Prospective cohort | PsA initiating TOF | 6 months | 51.2 |
| Campanilho Marques 2010 | Retrospective cohort | PsA on active TNFi treatment for more than 9 months | 9 months | 33.3 |
| Iervolino 2012 | Prospective observational | PsA initiating TNFi | 3 months | 6.8 |
| Bonafede 2012 | Retrospective cohort | PsA initiating ETN, ADA, INF | 12months | ETN:53, ADA:47, IFX:44 |
| Fagerli 2013 | Prospective observational | PsA starting 1st TNFi | 3 months | 21.6 |
| Galindez Agirregoikoa 2021 | Prospective observational | PsA starting TOF | 6 months | 23 |
| Takami 2023 | Retrospective cohort | PsA initiating ADA, CZP, SEC or IXE | 24 months | ADA: 24.0, CZP:4.8, SEC:6.2, IXE:8.2 |
| Pons 2023 (abstract) | Retrospective cohort | PsA patients from 12 countries with SEC initiation | 12months | 37 |
|  |  |  | 24 months | 51 |
| Erik 2023 (abstract) | Retrospective cohort | PsA initiating bDMARD | 12months | 59.7 |
|  |  |  | 24 months | 80.1 |

**Key**: ADA= adalimumab; ANK= anakinra; ASDAS= axial spondylarthritis disease activity score; bDMARD= biologic disease-modifying antirheumatic drug; CDAI= clinical disease activity index; CZP= certolizumab pegol; CRP= C-reactive protein; DAPSA= disease activity psoriatic arthritis; ETN= etanercept; GC= glucocorticoids; GOL= golimumab; GUS= guselkumab; IFX= infliximab; IL= interleukin; IXE= ixekizumab; LDA= low disease activity; MDA= minimal disease activity; MOA= mechanism of action; MODA= moderate disease activity; MSK= musculoskeletal; NA= not applicable; PASDAS= psoriatic arthritis disease activity score; PsA= psoriatic arthritis; QoL= quality of life; RIS= risankizumab; SEC= secukinumab; TOF= tofacitinib; TNF= tumour necrosis factor; UPA= upadacitinib; UST= ustekinumab; VLDA= very low disease activity. *All percentages are reported to 1 decimal place.

**Supplementary Table 6**. Summary of real-world studies reporting the percentage prevalence* of treatment discontinuation/switching according to different classes of biologics, at different timepoints.

|  | **TNFi** | **Other b/tsDMARDs** | **All b/tsDMARDs** |
| --- | --- | --- | --- |
| **At 3 months** | | | |
| **Treatment-IR** | 4.5-19.3 | 1.0-16.2 | 1.0-20.0 |
| **Treatment –naive** | 6.8-21.6 | NA | 6.8-21.6 |
| **At 6 months** | | | |
| **Treatment-IR** | 1.7-49.0 | 5.1-51.2 | 5.1-51.2 |
| **At 12 months** | | | |
| **Treatment-IR** | 2.7-73.9 | 6.2-71.8 | 2.7-73.9 |
| **Treatment –naive** | 2.7-24.5 | 13.0 | 2.7-55.5 |
| **At 24 months** | | | |
| **Treatment-IR** | 27.2-78.7 | 8.0-68.1 | 8.0-80.1 |
| **At 36 months** | | | |
| **Treatment-IR** | 44.0-84.9 | 24.0-87.7 | 44-84.9 |
| **At 96 months** | | | |
| **Treatment-IR** | 27.3-62.3 | NA | 27.3-62.3 |

Prevalences as stated in the table are given as a range of percentages to 1 decimal place accuracy. Note that the definition of treatment-IR varies between studies (shown elsewhere). **Key:** b/tsDMARDs= biologic/targeted synthetic disease modifying anti-rheumatic drugs; IR: inadequate responders; NA = not applicable; TNFi= tumour necrosis factor inhibitor. *All percentages are reported to 1 decimal place.

**Supplementary Table 7.** Summary of real-world studies reporting the percentage of patients having persistent or high disease activity despite treatment with b/tsDMARDs

| **First author and year** | **Type of study** | **Population included** | **Definition of D2M/TR PsA** | **Percentage treatment discontinuation of b/tsDMARDs** | |
| --- | --- | --- | --- | --- | --- |
|  |  |  |  | **Time point** | **Prevalence of D2M/TR PsA (mean/range, unless otherwise stated)*** |
| Iannone 2019 | Retrospective cohort | Starting CZP | DAPSA LDA/Remisson | 3 months | 52.8 |
| Iannone 2021 | Retrospective cohort | Starting GOL | CDAI-LDA | 6 months | 64.0 (bDMARD-naive)  61.0 (GOL as 2^nd^ line)  65.0 (GOL as 3^rd^ line) |
|  |  |  | CDAI-LDA | 12 months | 75.0 (bDMARD-naive)  71.0 (GOL as 2^nd^ line)  65.0 (GOL as 3^rd^ line) |
| Lindstrom 2021 | Prospective cohort | Starting SEC, ADA, CZP, ETN, GOL, IFX 2015-2018 | DAPSA LDA | 6 months | 1^st^ line bDMARD:  ADA: 72.0  SEC: 61.0  ETN: 69.0  IFX: 67.0  GOL:70.0  CZP: 74.0  2^nd^ line bDMARD:  ADA: 63.0  SEC: 43.0  ETN: 57.0  IFX: 68.0  GOL:55.0  CZP: 62.0  3^rd^ line bDMARD:  ADA: 49.0  SEC: 43.0  ETN: 46.0  IFX: 44.0  GOL 52.0  CZP: 41.0 |
|  |  |  | DAPSA Remission |  | 1^st^ line bDMARD:  ADA: 32.0  SEC: 20.0  ETN: 28.0  IFX: 22.0  GOL:23.0  CZP: 30.0  2^nd^ line bDMARD:  ADA: 17.0  SEC: 13.0  ETA: 16.0  IFX: 20.0  GOL:27.0  CZP: 20.0  3^rd^ line bDMARD:  ADA: 13.0  SEC: 8.0  ETN: 10.0  IFX: 7.0  GOL: 11.0  CZP: 3.0 |
| Gossec 2022 | Prospective cohort | Treated with UST or TNFi | cDAPSA LDA /remission | 12 months | UST: 55.9/ 22.1  TNFi: 67.1/ 31.7 |
|  |  |  | MDA/VLDA |  | TNFi: 43.1/ 12.6  UST: 34.2/ 11.9 |
| Gossec 2023 | Prospective cohort | Treated with UST or TNFi | cDAPSA LDA /remission | 3 years | UST: 58.6/ 31.4  TNFi: 69.8/ 45.0 |
|  |  |  | MDA/VLDA |  | UST: 41.4/ 19.2  TNFi: 54.2/ 26.9 |
| Glintborg 2011 | Prospective cohort | Treated with TNFi | EULAR good response | 6 months | 54.0 |
| Kristenser 2016 | Prospective cohort | Initiating TNFi | ACR20 | 3 months | 2^nd^ line b/tsDMARD: 49.0/ 39.0–59.0  3^rd^ line b/tsDMARD: 28.0/ 12.0–44.0 |
|  |  |  |  | 6 months | 2^nd^ line b/tsDMARD: 44.0/ 32.0–56.0  3^rd^ line b/tsDMARD: 33.0/ 11.0–55.0 |
|  |  |  | ACR50 | 3 months | 2^nd^ line b/tsDMARD: 22.0/ 14.0–30.0  3^rd^ line b/tsDMARD: 17.0/ 3.0–31.0 |
|  |  |  |  | 6 months | 2^nd^ line b/tsDMARD: 26.0/ 16.0–36.0  3^rd^ line b/tsDMARD: 6.0/ 0.0–17.0 |
|  |  |  | EULAR good response | 3 months | 2^nd^ line b/tsDMARD: 27.0/ 18.0–35.0  3^rd^ line b/tsDMARD: 13.0/ 1.0–25.0 |
|  |  |  |  | 6 months | 2^nd^ line b/tsDMARD: 31.0/ 20.0-42.0  3^rd^ line b/tsDMARD 19.0/ 1.0-37.0 |
| Iannone 2018 | Retrospective cohort | Starting UST | DAPSA remission | ,  6 months | bDMARD-naïve: 34.0  TNF-IR: 17.0 |
|  |  |  |  | 12 months | bDMARD-naïve: 34.0  TNF-IR: 15.0 |
|  |  |  | Lundex remission | 6 months | bDMARD naïve: 29.0  TNF-IR: 12.0 |
|  |  |  |  | 12months | bDMARD-naïve: 26.0  TNF-IR: 9.0 |
| Moreno-Ramos 2022 | Retrospective cohort | Starting SEC | DAS28-CRP remission (<2.6) | 2 years | 93% |
|  |  |  |  | 3 years | 93% |
|  |  |  | DAS28-CRP LDA (<3.2) | 2 years | 100% |
|  |  |  |  | 3 years | 100% |
| Gentiloni 2023 | Retrospective cohort | Treated with UPA | MDA | 6 months | 47.0 |
|  |  |  | DAPSA remission |  | 23.0 |
|  |  |  | ASDAS-CRP inactive disease |  | 8.0 |
| D’Angelo 2024 | Retrospective cohort | Starting GOL after 1 TNFi failure | MDA | 12 months | 37.1/ 95% CI: 25.2–50.3 |
|  |  |  | DAS28-CRP <2.6 |  | 72.7/ 95% CI:59–83.9 |
|  |  |  | Good/moderate EULAR response |  | 78.4/ 95% CI 61.8–90.2 |
| Lopalco 2024 (abstract) | Retrospective cohort | Treated with UPA | MDA | 12 months | 30.0 |
|  |  |  | VLDA |  | 20.0 |
| Pantano 2024 (abstract) | Retrospective cohort | Treated with RIS | DAPSA remission | 6 months | 19.0 |
|  |  |  | DAPSA-LDA |  | 43.0 |
| Ruscitti 2024 | Retrospective cohort | Starting GUS | DAPSA <14 | 3months | 29.7 |
|  |  |  |  | 6months | 39.6 |
| Fagerli 2013 | Prospective cohort | Starting 1st TNFi 2001- 2011 | EULAR good response | 3 months | 20.0 |

**Key**: ACR20/50 = American College of Rheumatology 20% or 50% response respectively; ADA= adalimumab; ASDAS= axial spondyloarthritis disease activity score; CDAI= clinical disease activity index; CI = confidence interval; CRP = C-reactive protein; CZP= certolizumab pegol; DAPSA= disease activity psoriatic arthritis; DAS28-CRP= disase activity score 28-joint count using CRP; ETN= etanercept; GOL= golimumab; GUS= guselkumab; IFX=infliximab; LDA= low disease activity; MDA= minimal disease activity; RIS= risankizumab; SEC= secukinumab; TNF= tumor necrosis factor; UPA= upadacitinib; UST= ustekinumab; VLDA= very low disease activity. *All percentages are reported to 1 decimal place.

# **Supplementary Table 8**: Summary of real-world studies reporting the percentage of patients having persistent or high disease activity despite treatment with b/tsDMARDs

|  | **MDA** | **VLDA** | **DAPSA-LDA** | **DAPSA-remission** | **EULAR moderate response** |
| --- | --- | --- | --- | --- | --- |
|  | **At 6 months** | | | | |
| TNF | - | - | 28%-59% | 68%-97% | 46 -81% |
| Other b/tsDMARDs | 53% | - | 39.0%-57.0% | 57%-92% |  |
|  | **At 12 months** | | | | |
| TNF | 56.9-62.9% | 87.4% | 32.9.7% | 68.3% | 21.6% |
| Other b/tsDMARDs | 65.8%-70.0% | 80.0%-88.1% | 44.1% | 66%-85% | - |
|  | **At 3 years** | | | | |
| TNF | 45.8% | 73.1% | 30.2% | 55.0% | - |
| Other b/tsDMARDs | 58.6% | 80.8% | 41.4% | 68.6% | - |

**Key:** b/tsDMARDs= biologic/targeted synthetic disease-modifying antirheumatic drugs; DAPSA= disease activity psoriatic arthritis; MDA= minimal disease activity; TNF= tumor necrosis factor; VLDA= very low disease activity.

# **Supplementary table 9:** Long term outcomes affected by the occurrence of D2M/TR PsA

| **First author and year** | **Outcome** | **Univariate analysis** | | | **Multivariate analysis** | | |
| --- | --- | --- | --- | --- | --- | --- | --- |
|  |  | **OR** | **95% CI** | **p value** | **OR/ β** | **95% CI** | **p value** |
| Snoeck Henkemans 2022 | HAQ DI | NA | NA | NA | Non sustained MDA β 0.31  No MDA β 0.81 | 0.19- 0.43  0.70- 0.92 | p <0.001  p <0.001 |
| Fagerli 2013 | HAQ-DI | NA | NA | Non-switchers vs 2^nd^ TNFi (switchers) p 0.01  1^st^ versus 2^nd^ TNFi (switchers)  p 0.11 | NA | NA | NA |
| Snoeck Henkemans 2022 | EQ-5D-5L | NA | NA | NA | Non sustained MDA β -0.07  No MDA β -0.23 | -0.12 to -0.03  -0.28 to -0.18 | p 0.48  p <0.001 |
| Gossec 2023 |  | UST 11.0  TNFi 18.9 | 6.5-15.4  14.0-23.9 | NA | NA | NA | NA |
| Snoeck Henkemans 2022 | PCS (SF-36) | NA | NA | NA | Non sustained MDA β -6.77  No MDA β -13.51 | -8.77 to -4.78  -15.41 to -11.62 | p <0.001  p <0.001 |
| Snoeck Henkemans 2022 | MCS (SF-36) | NA | NA | NA | Non sustained MDA β -3.07  No MDA β -7.51 | -5.64 to -0.50  -9.95 to -5.08 | p 0.37  p <0.001 |
| Snoeck Henkemans 2022 | VAS | NA | NA | NA | Non sustained MDA β 15.8  No MDA β 35.38 | 10.71 to 20.89  30.57 to 40.18 | p <0.001  p <0.001 |
| Snoeck Henkemans 2022 | BRAF-MDQ | NA | NA | NA | Non sustained MDA β 7.87  No MDA β 17.88 | 4.40 to 11.33  14.60 to 21.16 | p <0.001  p <0.001 |
| Gossec 2023 | WPAI | Absenteeism UST −11.8 and TNFi −20.8  Presenteeism UST −21.6 and TNFi −37.3  Work productivity loss UST −24.9 and TNFi −44.5  Activity impairment UST −28.0 and TNFi −40.7 | UST −18.4 to −5.1  TNFi −27.9 to −13.6  UST −28.3 to −14.8  TNFi −42.8 to −31.8  UST −34.0 to −15.8  TNFi −50.6 to −38.4  UST −32.4 to −23.5  TNFi −44.5 to −36.8 | NA | NA | NA | NA |
| Mease 2023 (abstract) | WPAI | Reduction of WPAI at week 16: 15.6% BIME vs 3.6% PBO  Improvement in work productivity at week 40: 21.5% BIME vs. 21.6% PBO | NA | NA | NA | NA | NA |
| Snoeck Henkemans 2022 | HADS | NA | NA | NA | Anxiety  Non sustained MDA β 1.34  No MDA β 3.26  Depression  Non sustained MDA β 1.30  No MDA β 4.04 | 0.26 to 2.42  2.25 to 4.27  0.30 to 2.30  3.10 to 4.99 | p 0.28  p <0.001  p 0.21  p <0.001 |
| Fagerli 2013 | PhGA | NA | NA | Non-switchers vs 2^nd^ TNFi (switchers)  p <0.001  1^st^ versus 2^nd^ TNFi (switchers)  p 0.77 | NA | NA | NA |
| Fagerli 2013 | PtGA | NA | NA | Non-switchers vs 2^nd^ TNFi (switchers) p 0.03  1^st^ versus 2^nd^ TNFi (switchers)  p 0.05 | NA | NA | NA |
| Gossec 2023 | PsAID12 | PsAID-12: overall  UST −2.9 and TNFi -3.5  PsAID-12: pain  UST – and TNFi −3.8  PsAID-12: skin  UST −3.9 and TNFi −3.1 | UST −3.2−2.5  TNFi −3.9−3.2  UST −3.3−2.5  TNFi −4.2−3.4  UST −4.4−3.4  TNFi −3.6−2.7 | NA | NA | NA | NA |

**Key:** BRAF-MDQ= Bristol rheumatoid arthritis fatigue multidimensional questionnaire; CI= confidence interval; EQ-5D-5L= EuroQoL 5-dimensaion 5-level; HADS= hospital anxiety and depression scale; HAQ-DI= health assessment questionnaise disability index; MCS= mental component summary; MDA = minimal disease activity; NA= not applicable; OR= odds ratio; PCS= physical component summary; PhGA= physician global assessment of disease activity; PsAID12= psoriatic arthritis impact of disease; PtGA= patient global assessment of disease activity; SF-36= short form 36; TNFi= tumour necrosis factor inhibitor; UST= ustekinumab; VAS= visual analogue scale; WPAI= work productivity and activity impairment; β = beta statistic;

# **Supplementary Table 10**. Study characteristics for all studies included in PICO3

| **First author and year** | **Study design** | **Definition of D2M/TR** | **No cases** | **No controls** | **RoB** |
| --- | --- | --- | --- | --- | --- |
| Alp 2024 | Cohort | Multi-domain definition: patients must meet all of the following criteria (A/B/C):  A. Discontinuation ≥1 csDMARDs  B. ≥2b/tsDMARDs ≥2 MOA  C. DAPSA ≥14 | 33 | 138 | Mod |
| Roseman 2024 | Cohort | Two definitions tested by the authors (d1/d2) were:  (d1) Unacceptable pain on the PASS scale with VAS pain >40mm  (d2) Refractory pain: Unacceptable pain + poorly controlled inflammation | 61 | - | Mod |
| Samuel 2024 | Cohort | Single-item patient questionnaire designed by authors containing a range of disease-specific measures | 62 | 116 | Mod |
| Vassilakis 2024 | Cohort | Multi-domain definition. Patients had to fulfil all criteria below (A to F):  A. ≥6m disease duration  B. Tx. discont. ≥1 csDMARD  C. Discontinuation ≥2 b/tsDMARDs, ≥2 MOA (excluding apremilast)  D. ≥1 of the following  E. DAPSA ≥14  F> Not meeting MDA criteria | 77 | 399 | Mod |
| Dalen 2023 | Cohort | Time to discontinuation 1^st^ b/tsDMARD, defined as one of the following (A/B/C):  A. Absence of any repeat b/tsDMARD prescriptions  B. Having had a treatment gap of >60 days  C. Filing a prescription for a different b/tsDMARD | Av. time to LOR* | Av. time to LOR* | Mod |
| Gentiloni 2023 | Cohort | Two definitions tested by authors were (d1/d2):  (d1) Discontinuation 1^st^ line TNFi where exposed to ≥1 csDMARDs  (d2) Discontinuation 2^nd^ line + b/tsDMARD | 35 | 31 | Mod |
| Joven 2023 | Cohort | Time to discontinuation IXE at 24 wks, any line of Tx | 28 | 61 | Mod |
| Philippoteaux 2023 | Cohort | Two definitions (d1/d2) tested by authors were:  (d1) D2T PsA: discontinuation ≥2 b/tsDMARDs with ≥2 MOA  (d2) Very D2T PsA: discontinuation ≥2 b/tsDMARDs with ≥2 MOA AND this happened <2yrs | 49 | 101 | Mod |
| Rida 2023 | Cohort | Two definitions (d1/d2) tested by the authors were  (d1) Discontinuation 1^st^ line b/tsDMARD  (d2) Discontinuation 2^nd^ + line b/tsDMARD | 254 | - | Mod |
| Hunter 2022 | Cohort | Multi-domain definition: patients reporting ≥1 of (A to F) any b/tsDMARD, any line of treatment:  A. <80% adherence to therapy  B. Tx switch  C. Add-in therapy  D. ≥ GC injections  E. Start/ increase in PO GC  F. Add in topical Tx for PsO | 1103 | 330 | Mod |
| Mateo-Soria 2022 | Cohort | Time to discontinuation of any line of b/tsDMARD | Av. Time to LOR* | Av. Time to LOR* | Mod |
| Perrotta 2022 | Cohort | Multi-domain definition defined as patients meeting ALL THREE criteria (A to C)  A. LOR to ≥2 b/tsDMARDs with ≥2 MOA according to EULAR/GRAPPA definition of treatment failure AND failing ≥1 csDMARD.  B. Active PsA: ≥1 of  i. DAPSA ≥14  ii. Not achieving MDA  iii. Biochemical/ imaging parameters of active PsA  iv. Rapid radiographic progression  v. Not meeting criteria i-iv above but still have PsA symptoms that were significantly impacting QoL  C. Management of signs/ symptoms perceived as problematic by the rheumatologist and/ or patient | 36 | 70 | Mod |
| Iannone 2021 | Cohort | Time to discontinuation 1^st^ line b/tsDMARD  Time to discontinuation ≥2 lines of b/tsDMARD any MOA | 153  144 | 255  255 | Mod |
| Lorenzin 2021 | Cohort | Discontinuation any b/ts-DMARD, any line of Tx | 117 | 129 | Mod |
| Vieira-Sousa 2020 | Cohort | Discontinuation 1^st^ b/tsDMARD | 269 | 481 | Mod |
| Tsuruta 2019 | Cohort | Discontinuation any b/tsDMARD, any line of Tx | 47 | 105 | Mod |
| Stober 2018 | Cohort | Authors tested two definitions (d1/d2) of D2T PsA:  (d1) Discontinuation ≥2csDMARDs and 1^st^ b/tsDMARD  (d2) Discontinuation ≥2csDMARDs and ≥2b/tsDMARDs any MOA | Av. Time to LOR* | Av. Time to LOR* | Mod |
| Harrold 2017 | Cohort | Time to discontinuation 1^st^ b/tsDMARD | Av. Time to LOR* | Av. Time to LOR* | Mod |
| Glintborg 2011 | Cohort | Time to discontinuation 1^st^ b/tsDMARD | Av. Time to LOR* | Av. Time to LOR* | Mod |
| Saad 2009 | Cohort | Time to discontinuation 1^st^ b/tsDMARD | Av. Time to LOR* | Av. Time to LOR* | Mod |
| Ferreira 2024 (abstract) | Cohort | Time to discontinuation 1^st^ b/tsDMARD | 28 | 55 | NA |
| Harrison 2024 (abstract) | Cohort | Patients must fulfil criteria A and B (below):   1. Discontinuation ≥2 b/tsDMARDs, any MOA 2. DAPSA ≥14 | 91 | 42 | NA |
| Kalyoncu 2024 (abstract) | Cohort | Discontinuation ≥2 b/tsDMARDs, ≥2 MOA | 2,605 | 9,220 | NA |
| Cincinelli 2023 (abstract) | Cohort | Patients must fulfil criteria A and B (below):   1. Discontinuation ≥2 b/tsDMARDs, ≥2 MOA AND discontinuation ≥1 cs-DMARD 2. Active PsA (≥1 from the list below):   i. DAPSA ≥14  ii. Not meeting MDA  iii. Any other clinical signs/ symptoms, rapid radiographic progression or reduced QoL attributed to PsA  iv. PsA management perceived as problematic by rheumatologist and/or patient. | 8 | 261 | NA |
| Rodriguez-Laguna 2023 (abstract) | Cohort | Discontinuation 1^st^ b/tsDMARD | 109 | 31 | NA |

**Key**: Av. time to LOR= average time to lack or loss of response; b/tsDMARD= biologic or targeted synthetic DMARD; csDMARD= conventional synthetic disease-modifying anti-rheumatic drug; d = definition; DAPSA = disease activity score PsA. MDA= minimal disease activity; MOA= mechanism of action; Mod = moderate; NA = not applicable; QoL = quality of life; RoB= Risk of Bias; VAS= visual analogue scale.

# **Supplementary Table 11**. Population baseline characteristics of studies included in PICO3.

| **First author and year** | **Male sex (n/ %)** | **BMI* (mean/SD)** | **PsA disease duration in years* (mean /SD)** | **Age at study entry in years (mean/SD)*** | **PsO ever, (n/%)** | **IBD ever (n/%)** | **Uveitis ever (n/%)** | **Axial involvement ever (n/%)** |
| --- | --- | --- | --- | --- | --- | --- | --- | --- |
| Alp 2024 | 55/ 32.2 | 29.2/ 5 | NA | 48.16/ 11.2 | NA | NA | NA | 61/ 35.7 |
| Roseman 2024 | 181/ 52 | NA | 10/ 9 | 47/ 12 | NA | NA | NA | NA |
| Samuel 2024 | 85/ 49.1 | 31.3/ 7.2 | NA | 51.7/ 13.5 | NA | NA | NA | NA |
| Vassilakis 2024 | NA | NA | NA | NA | NA | NA | NA | NA |
| Dalen 2023 | 1737/ 46.6 | NA | NA | 49.3/ 12.8 | NA | NA | NA | NA |
| Gentiloni 2023 | 40/ 31.7 | 26.7/ 5.1 | NA | 56.5/ 11.4 | 78/ 61.9 | 6/ 4.8 | 6/ 4.8 | 54/ 42.9 |
| Joven 2023 | 40/ 44.9 | NA | NA | 51.5/ 11.6 | 75 | NA | NA | 12/ 13.6 |
| Philippoteaux 2023 | NA | NA | NA | NA | NA | NA | NA | NA |
| Rida 2023 | 301/ 57.7 | NA | NA | 48.2/ 13 | NA | NA | NA | 186/ 37.6 |
| Hunter 2022 | 678/ 47.3 | NA | NA | 49.5/ 11.2 | NA | NA | NA | NA |
| Mateo-Soria 2022 | 79/ 49.7 | NA | NA | NA | 131/85.6 | NA | NA | 9/ 6 |
| Perrotta 2022 | NA | NA | NA | NA | NA | NA | NA | NA |
| Iannone 2021 | 192/ 34.8 | 26.8/ 5 | 7.8/7 | 51/ 11 | NA | 23/ 4.2 | 8/1.4 | NA |
| Lorenzin 2021 | NA | NA | NA | NA | NA | NA | NA | NA |
| Vieira-Sousa 2020 | 373/ 49.7 | NA | 6.6/ 6.8 | 47.6/ 11.6 | NA | NA | NA | NA |
| Tsuruta 2019 | 204/ 71.6 | NA | NA | NA | 35 | NA | NA | 62/ 21.8 |
| Stober 2018 | 92/ 49 | 32.6/ 7.4 | NA | 47.2/ 11.4 | NA | NA | NA | NA |
| Harrold 2017 | 293/ 42.3 | NA | 9.5/8.4 | 50.8/ 11.6 | NA | NA | NA | NA |
| Glintborg 2011 | 368/ 48.2 | NA | NA | NA | NA | NA | NA | NA |
| Saad 2009 | 266/ 47.7 | NA | 12.4/8.7 | 45.7/ 11.1 | NA | NA | NA | NA |
| Ferreira 2024 (abstract) | 43/ 51.8 | NA | NA | 53.2/ 11.8 | NA | NA | NA | NA |
| Harrison 2024 (abstract) | 61/ 45.9 | 32/ 7.5 | 13/ 7.9 | 52.3/ 12.8 | NA | 10/ 7.5 | 13/ 9.8 | 32.3/ 43 |
| Kalyoncu 2024 (abstract) | NA | NA | NA | NA | NA | NA | NA | NA |
| Cincinelli 2023 (abstract) | 140/ 52 | NA | NA | 52.6/ 11.9 | 205/ 76.2 | 4/ 1.5 | 6/ 2.2 | 38/ 14.1 |
| Rodriguez-Laguna 2023 (abstract) | 67/ 47.5 | NA | NA | 48.1/ 13.2 | 108/ 76.6 | NA | NA | 22/ 15.6 |

**Key**: *Values for numeric continuous variables are expressed as either mean (standard deviation (SD)) or median (Interquartile range (IQR)) as per the original manuscript; data missing or not reported; n= number; SD = standard deviation; PsO = psoriasis (skin); IBD = inflammatory bowel disease

# **Supplementary Table 12.** Summary of all predictors of D2T PsA tested in PICO3 studies, including the number of statistical tests for each predictor and the percentage of positive associations with D2T PsA

| **Predictor** | **No. tests for this variable overall** | **No. tests that report a positive association with D2T PsA (p<0.05)** | | **No. tests that report a negative association with D2T PsA (p<0.05)** | | **Borderline association (p=0.5-1.0)** | **Percentage of positive associations overall (%)** |
| --- | --- | --- | --- | --- | --- | --- | --- |
|  |  | **Multivariate statistical model** | **Univariate statistical model** | **Multivariate statistical model** | **Univariate statistical model** | **Uni- or multi-variate statistical model** |  |
| Sex (female) | 49 | 10 | 10 | 14 | 14 | 1 | 41.7 |
| Age - younger age at study enrolment (years) | 39 | 2 | 2 | 16 | 16 | 3 | 11.1 |
| BMI (high vs low)* | 31 | 5 | 3 | 11 | 9 | 3 | 28.6 |
| csDMARD* | 26 | 3 | 5 | 2 | 13 | 3 | 34.8 |
| Comorbidities - PsO (skin)* | 23 | 3 | 8 | 5 | 1 | 6 | 64.7 |
| Disease activity - Dactylitis* | 23 | 0 | 2 | 7 | 13 | 1 | 9.1 |
| Clinical - SJC (higher) | 21 | 9 | 4 | 2 | 5 | 1 | 65.0 |
| Duration PsA (years) | 20 | 2 | 4 | 2 | 12 | 0 | 30.0 |
| Disease activity - Enthesitis* | 20 | 2 | 2 | 6 | 9 | 1 | 21.1 |
| Clinical - TJC (higher) | 19 | 4 | 6 | 2 | 6 | 1 | 55.6 |
| b/tsDMARD - use ever* | 17 | 5 | 4 | 4 | 3 | 1 | 56.3 |
| Co-morbidities - anxiety/depression^ | 17 | 5 | 5 | 4 | 3 | 0 | 58.8 |
| Bloods - CRP* | 17 | 1 | 4 | 2 | 10 | 0 | 29.4 |
| Smoking* | 15 | 1 | 2 | 5 | 7 | 0 | 20.0 |
| Family history* | 13 | 0 | 0 | 0 | 13 | 0 | 0.0 |
| Comorbidities - other | 12 | 2 | 3 | 0 | 7 | 0 | 41.7 |
| Comorbidities - chronic pain& | 12 | 1 | 6 | 4 | 1 | 0 | 58.3 |
| Bloods - ESR* | 12 | 0 | 3 | 2 | 7 | 0 | 25.0 |
| b/tsDMARD - year started* | 11 | 2 | 0 | 3 | 6 | 0 | 18.2 |
| Comorbidities - CVD% | 10 | 0 | 1 | 2 | 7 | 0 | 10.0 |
| Disease activity - DAPSA* | 10 | 1 | 4 | 3 | 2 | 0 | 50.0 |
| Joints - peripheral, any distribution* | 10 | 1 | 1 | 2 | 6 | 0 | 20.0 |
| Comorbidities - PsO (nail)* | 9 | 1 | 3 | 4 | 1 | 0 | 44.4 |
| EMMs - IBD* | 9 | 1 | 2 | 0 | 6 | 0 | 33.3 |
| EMMs - Uveitis* | 9 | 0 | 1 | 0 | 8 | 0 | 11.1 |
| Glucocorticosteroids * | 9 | 1 | 3 | 2 | 3 | 0 | 44.4 |
| Joints - axial* | 9 | 1 | 6 | 1 | 1 | 0 | 77.8 |
| PROs - Pt VAS pain (higher, 0-100) | 9 | 2 | 6 | 1 | 0 | 0 | 88.9 |
| Comorbidities - multimorbidity@ | 8 | 1 | 3 | 3 | 1 | 0 | 50.0 |
| Employment status* | 8 | 0 | 3 | 4 | 1 | 0 | 37.5 |
| Disease activity - DAS-28, higher | 7 | 1 | 0 | 1 | 5 | 0 | 14.3 |
| Disease activity – PhLDA (higher, range 1-5) | 7 | 1 | 2 | 1 | 2 | 1 | 50.0 |
| Ethnicity* | 7 | 0 | 0 | 1 | 6 | 0 | 0.0 |
| PROs - CDAI* | 6 | 4 | 0 | 0 | 2 | 0 | 66.7 |
| PROs - Pt VAS global (higher, 0-100) | 6 | 3 | 3 | 0 | 0 | 0 | 100.0 |
| Age - at PsA onset (years) | 6 | 0 | 1 | 1 | 3 | 1 | 20.0 |
| Educational attainment* | 5 | 1 | 0 | 0 | 4 | 0 | 20.0 |
| Radiographic damage* | 5 | 1 | 0 | 0 | 4 | 0 | 20.0 |
| Age - at 1st b/ts-DMARD (years) | 4 | 2 | 1 | 0 | 1 | 0 | 75.0 |
| Bloods - HLA-B27 positive (Y/N) | 4 | 1 | 1 | 0 | 1 | 1 | 66.7 |
| PROs - EQ-5D (high vs. low) | 4 | 2 | 2 | 0 | 0 | 0 | 100.0 |
| PROs - PROMIS SF8 fatigue (high vs. low) | 4 | 3 | 1 | 0 | 0 | 0 | 100.0 |
| PROs - PROMIS SF8 pain (high vs. low) | 4 | 3 | 1 | 0 | 0 | 0 | 100.0 |
| PROs - PROMIS-29 physical function (high vs. low) | 4 | 3 | 1 | 0 | 0 | 0 | 100.0 |
| PROs - PtLDA (high vs. low) | 4 | 0 | 2 | 1 | 1 | 0 | 50.0 |
| Comorbidities - OA£ | 3 | 0 | 2 | 0 | 1 | 0 | 66.7 |
| Disease activity - MDA* | 3 | 1 | 1 | 0 | 1 | 0 | 66.7 |
| NSAIDs* | 3 | 0 | 0 | 0 | 3 | 0 | 0.0 |
| PROs - EGA (high vs. low) | 3 | 1 | 1 | 0 | 1 | 0 | 66.7 |
| Analgesics* | 2 | 0 | 0 | 0 | 2 | 0 | 0.0 |
| BASDAI (high vs. low)* | 2 | 0 | 0 | 1 | 1 | 0 | 0.0 |
| Bloods - ANA positive ever (Y/N) | 2 | 0 | 0 | 0 | 2 | 0 | 0.0 |
| Bloods - CCP positive ever (Y/N) | 2 | 0 | 0 | 0 | 2 | 0 | 0.0 |
| Clinical - PASI* | 2 | 0 | 0 | 0 | 2 | 0 | 0.0 |
| Duration PsO (years) | 2 | 0 | 1 | 1 | 0 | 0 | 50.0 |
| EMMs* | 2 | 0 | 0 | 0 | 2 | 0 | 0.0 |
| PROs - HAQDI (high vs. low) | 2 | 0 | 2 | 0 | 0 | 0 | 100.0 |
| PROs - PASS (Y vs N) | 2 | 0 | 2 | 0 | 0 | 0 | 100.0 |
| PROs - Patient reported pain (0-10) | 2 | 0 | 0 | 2 | 0 | 0 | 0.0 |
| PROs - PROMIS SF8 ability to participate (high vs. low) | 2 | 1 | 1 | 0 | 0 | 0 | 100.0 |
| PROs - PROMIS-29 anxiety (high vs. low) | 2 | 1 | 1 | 0 | 0 | 0 | 100.0 |
| PROs - PROMIS-29 depression (high vs. low) | 2 | 0 | 1 | 1 | 0 | 0 | 50.0 |
| PROs - PROMIS-29 sleep (high vs. low) | 2 | 1 | 1 | 0 | 0 | 0 | 100.0 |
| Age - at PsO onset (years) | 1 | 0 | 0 | 0 | 1 | 0 | 0.0 |
| ASDAS (high vs. low) | 1 | 0 | 0 | 0 | 1 | 0 | 0.0 |
| b/ts-DMARD - discont, poor dermatological control | 1 | 1 | 0 | 0 | 0 | 0 | 100.0 |
| b/ts-DMARD - time diagnosis to bDMARD1 (years or months) | 1 | 0 | 1 | 0 | 0 | 0 | 100.0 |
| Bloods - RF positive ever (Y vs. N) | 1 | 0 | 0 | 0 | 1 | 0 | 0.0 |
| Clinical - EMS (minutes) | 1 | 0 | 1 | 0 | 0 | 0 | 100.0 |
| ETOH* | 1 | 0 | 0 | 0 | 1 | 0 | 0.0 |
| IP stay (ANY reason) | 1 | 1 | 0 | 0 | 0 | 0 | 100.0 |
| IP stay (for IA) | 1 | 1 | 0 | 0 | 0 | 0 | 100.0 |
| Marital status (Y vs. N) | 1 | 1 | 0 | 0 | 0 | 0 | 100.0 |
| More than 1 OPD <1yr starting TNFi (any specialty) | 1 | 1 | 0 | 0 | 0 | 0 | 100.0 |
| PROs - Extent impact ADLs (0-10) | 1 | 0 | 1 | 0 | 0 | 0 | 100.0 |
| PROs - PsA PtG PsO (1-5) | 1 | 1 | 0 | 0 | 0 | 0 | 100.0 |
| PROs - PsAID (median) | 1 | 0 | 1 | 0 | 0 | 0 | 100.0 |
| PsA lag symptoms to diagnosis | 1 | 0 | 0 | 0 | 1 | 0 | 0.0 |

**Legend**: Summary table of all the predictors of D2M/TR PsA for PICO3 studies. Reported as the total number of statistical test and percentage positive associations for each predictor with the outcome of interest (D2M/TR). Percentages are reported to 1 decimal place.

**Key**: *=authors may use slightly different variations to define this particular variable, but they are grouped here to provide an overall summary for the reader. Details of the exact definitions for each study can be found in the primary research papers; ANA=anti-nuclear antibodies; b/tsDMARD=biologic or targeted synthetic disease modifying anti-rheumatic drug; BASDAI=bath ankylosing spondylitis disease activity index; BMI=body mass index; CCP=c-citrullinate peptide antibodies; CDAI=clinical disease activity index; CI=confidence interval; CRP=C-reactive protein ; csDMARD=conventional synthetic disease modifying anti-rheumatic drug; CVD=cardiovascular disease; D2T PsA=difficult-to-treat PsA (as defined by the study authors); DAPSA=Disease activity score psoriatic arthritis; DAS-28=Disease activity score 28 joint count; EGA=evaluators global assessment; EMMs=extra-musculoskeletal manifestations; EMS=early morning stiffness; ETOH=alcohol; HAQDI=health assessment questionnaire disability index; HLA-B27=human leucocyte antigen B27; HR=hazard ratio; IBD=Inflammatory bowel disease; IP=inpatient; MDA=minimal disease activity; NA=not applicable/ missing; No.=number; NSAIDs=non-steroidal anti-inflammatory drugs; OA=osteoarthritis; OR=odds ratio; p val=p value; PASI=psoriasis assessment and severity index; PhG =physician global assessment; PhLDA=physician likert disease activity; PROMIS =patient reported outcome measurement information system; PROs=patient reported outcomes; PsA=psoriatic arthritis; PsAID=Psoriatic arthritis impact of disease; PsO=psoriasis; Pt=patient; PtG=patient global assessment; PtLDA=patient likert disease activity; RR=risk ratio; SJC=swollen joint count 66; TJC=tender joint count 68; TNFi=TNF inhibitor; VAS=visual analogue scale; VLDA=very low disease activity.

# **Supplementary Table 13.** Summary of odds/risk/hazard ratio and confidence interval/p values for each study that explored sex as a predictor of D2M/TR PsA

| **Sex (female vs. male)** | | | | | | | | |
| --- | --- | --- | --- | --- | --- | --- | --- | --- |
|  |  |  | **Multivariate** | | | **Univariate** | | |
| **Author and year** | **Group (G1-G6)** | **NOS ROB score** | **OR/RR/HR** | **95% CI** | **p val** | **OR/RR/HR** | **95% CI** | **p val** |
| Dalen 2023 | G1 | 6 | 0.866 | NA | <0.0001 | NA | NA | NA |
| Glintborg 2011 | G1 | 6 | 1.42 | 1.11-1.80 | 0.005 | 1.65 | 1.33-2.05 | <0.001 |
| Harrold 2017 (d1) | G1 | 6 | 1 | 0.65-1.53 | >0.05 | NA | NA | NA |
| Harrold 2017 (d2) | G2 | 6 | 1.22 | 0.91-1.63 | >0.05 | NA | NA | NA |
| Hunter 2022 | G3 | 6 | 1.37 | 1.06-1.78 | 0.0169 | NA | NA | 0.0007 |
| Iannone 2021 (d1) | G1 | 6 | NA | NA | NA | NA | NA | >0.05 |
| Iannone 2021 (d2) | G2 | 6 | NA | NA | NA | NA | NA | >0.05 |
| Joven 2023 | G3 | 5 | NA | NA | NA | 1.42 | 0.67-3.05 | 0.362 |
| Lorenzin 2021 (d1) | G2 | 6 | 2.38 | 1.49-3.81 | <0.001 | NA | NA | NA |
| Genteloni 2023 | G2, G4 | 5 | NA | NA | 0.123 | 2.537 | 1.03-6.25 | 0.043 |
| Perrota 2022 | G1, G4, G6 | 6 | NA | NA | NA | NA | NA | 0.37 |
| Rida 2023 (d1) | G3 | 5 | NA | NA | >0.05 | NA | NA | NA |
| Rida 2023 (d2) | G1 | 5 | NA | NA | >0.05 | NA | NA | NA |
| Rida 2023 (d3) | G2 | 5 | 0.12 | 0.02-0.69 | 0.018 | NA | NA | NA |
| Roseman 2024 (d1) | G5 | 5 | NA | NA | NA | 1.38 | 0.75-2.55 | 0.3 |
| Roseman 2024 (d2) | G6 | 5 | NA | NA | NA | 1.34 | 0.65-2.77 | 0.43 |
| Saad 2009 (d1) | G3 | 6 | 1.38 | 1.12-1.70 | <0.05 | 1.29 | 1.01-1.65 | <0.05 |
| Samuel 2024 (m1) | G6 | 5 | NA | NA | NA | NA | NA | 0.017 |
| Samuel 2024 (m2) | G6 | 5 | 1.11 | 0.26-3.15 | 0.87 | NA | NA | NA |
| Samuel 2024 (m3) | G6 | 5 | 1.32 | 0.23-2.52 | 0.66 | NA | NA | NA |
| Samuel 2024 (m4) | G6 | 5 | 1.22 | 0.25-2.67 | 0.74 | NA | NA | NA |
| Samuel 2024 (m5) | G6 | 5 | 1.27 | 0.22-2.82 | 0.72 | NA | NA | NA |
| Samuel 2024 (m6) | G6 | 5 | 1.52 | 0.20-2.23 | 0.51 | NA | NA | NA |
| Samuel 2024 (m7) | G6 | 5 | 1.75 | 0.21-2.37 | 0.57 | NA | NA | NA |
| Vieira-Sousa 2020 | G3 | 6 | 2.1 | 1.29-3.41 | 0.003 | NA | NA | NA |
| Mateo-Soria 2022 | G2 | 5 | 0.04 | 0.01-0.83 | 0.037 | 1.66 | 0.72-3.85 | 0.236 |
| Stober 2018 (d1) | G2 | 5 | 2.57 | 1.26-5.24 | 0.01 | 1.84 | 1.11-3.04 | 0.02 |
| Stober 2018 (d2) | G3 | 5 | NA | NA | NA | 1.69 | 0.60-4.76 | 0.32 |
| Tsurata 2019 | G2 | 6 | 0.93 | 0.39-2.17 | 0.86 | NA | NA | 0.33 |
| Alp 2024 | G1, G4 | 6 | NA | NA | NA | NA | NA | 0.878 |
| Vassilaklis 2024 (d1) | G2, G4 | 6 | NA | NA | >0.05 | NA | NA | 0.022 |
| Vassilaklis 2024 (d2) | G2, G4 | 6 | 3.03 | 1.08-8.47 | 0.034 | NA | NA | 0.001 |
| Vassilaklis 2024 (d3) | G2, G4 | 6 | NA | NA | >0.05 | NA | NA | 0.064 |
| Rodriguez-Laguna 2023 (EULAR poster) | G3 | 5 | NA | NA | NA | 18.28 | 14.66-22.79 | <0.05 |
| Cincinelli 2023 (EULAR oral) | G1, G4, G6 | 5 | NA | NA | NA | NA | NA | >0.05 |
| Ferreira 2024 (EULAR oral) | G3 | 6 | NA | NA | NA | NA | NA | 0.485 |
| Harrison 2024 (EULAR poster) | G2, G4 | 6 | NA | NA | NA | NA | NA | >0.2 |
| Kalyoncu 2024 (EULAR oral) | G1 | 6 | NA | NA | NA | 1.19 | 1.09-1.31 | <0.05 |

Table summarising the results of univariate and multivariate tests for this predictor across all studies included in PICO3. The definitions provided by the authors for D2M/TR PsA are summarised into groups, G1-G6, defined as follows**:** G1=loss/ lack of response to 1st b/tsDMARD; G2=loss/ lack of response to ≥2 b/tsDMARDs, with ≥2 mechanisms of action (MOA); G3=loss/ lack of response to b/tsDMARDs, mechanism and line of treatment not specified; G4=failure to achieve MDA by any composite measure ; G5=objective inflammation (SJC ≥3 OR raised CRP/ESR); G6=Patient/physician perception

**Key:** CI=confidence interval; d=definition, in the event that authors tested different definition of D2M/TR PsA in their paper; HR=hazard ratio; m=refers to different statistical models, in the event that the authors applied multiple statistical models to the same data; NA=not applicable/ missing; NOS ROB=Newcastle-Ottawa Scale Risk of Bias Score; OR=odds ratio; p val=p value; RR=risk ratio.

# **Supplementary Table 14**. Summary of odds/risk/hazard ratio and confidence interval/p values for each study that explored Age as a predictor of D2M/TR PsA

| **Age (younger)** | | | | | | | | |
| --- | --- | --- | --- | --- | --- | --- | --- | --- |
|  |  |  | **Multivariate** | | | **Univariate** | | |
| **Author and year** | **Group (G1-G6)** | **NOS ROB score** | **OR/RR/HR** | **95% CI** | **p val** | **OR/RR/HR** | **95% CI** | **p val** |
| Dalen 2023 | G1 | 6 | NA | NA | 0.5082 | NA | NA | 0.3798 |
| Glintborg 2011 | G1 | 6 | 1 | 0.99-1.01 | 0.61 | NA | NA | NA |
| Harrold 2017 (d1) | G1 | 6 | 1 | 0.99-1.02 | >0.05 | NA | NA | NA |
| Harrold 2017 (d2) | G2 | 6 | 1 | 0.99-1.01 | >0.05 | NA | NA | NA |
| Hunter 2022 | G3 | 6 | 1.04 | 0.65-1.67 | 0.8794 | NA | NA | 0.3736 |
| Iannone 2021 (d1) | G1 | 6 | NA | NA | NA | NA | NA | >0.05 |
| Iannone 2021 (d2) | G2 | 6 | NA | NA | NA | NA | NA | >0.05 |
| Joven 2023 | G3 | 5 | NA | NA | NA | 0.89 | 0.27-2.95 | 0.847 |
| Lorenzin 2021 (d1) | G2 | 6 | 1.01 | 0.99-1.03 | 0.322 | NA | NA | NA |
| Perrota 2022 | G1, G4, G6 | 6 | NA | NA | NA | NA | NA | 0.675 |
| Roseman 2024 (d1) | G5 | 5 | NA | NA | NA | 1.03 | 0.80-1.32 | 0.81 |
| Roseman 2024 (d2) | G6 | 5 | NA | NA | NA | 1.04 | 0.77-1.40 | 0.8 |
| Saad 2009 (d1) | G3 | 6 | 0.98 | 0.98-1.00 | >0.05 | 0.99 | 0.98-1.00 | >0.05 |
| Samuel 2024 (m1) | G6 | 5 | NA | NA | NA | NA | NA | 0.84 |
| Samuel 2024 (m2) | G6 | 5 | 1.01 | 0.97-1.06 | 0.57 | NA | NA | NA |
| Samuel 2024 (m3) | G6 | 5 | 1.03 | 0.98-1.08 | 0.26 | NA | NA | NA |
| Samuel 2024 (m4) | G6 | 5 | 1.01 | 0.97-1.06 | 0.63 | NA | NA | NA |
| Samuel 2024 (m5) | G6 | 5 | 1 | 0.96-1.05 | 0.86 | NA | NA | NA |
| Samuel 2024 (m6) | G6 | 5 | 1.01 | 0.97-1.06 | 0.61 | NA | NA | NA |
| Samuel 2024 (m7) | G6 | 5 | 1 | 0.96-1.04 | 0.9 | NA | NA | NA |
| Stober 2018 (d1) | G2 | 5 | 0.94 | 0.88-1 | 0.05 | 1 | 0.98-1.03 | 0.21 |
| Stober 2018 (d2) | G3 | 5 | NA | NA | NA | 1.06 | 1.01-1.11 | 0.02 |
| Tsurata 2019 | G2 | 6 | 3.65 | 1.62-8.23 | <0.01 | NA | NA | 0.04 |
| Alp 2024 | G1, G4 | 6 | NA | NA | P>0.05 | NA | NA | 0.072 |
| Vassilaklis 2024 (d1) | G2, G4 | 6 | NA | NA | >0.05 | NA | NA | 0.129 |
| Vassilaklis 2024 (d2) | G2, G4 | 6 | NA | NA | >0.05 | NA | NA | 0.211 |
| Vassilaklis 2024 (d3) | G2, G4 | 6 | NA | NA | >0.05 | NA | NA | 0.056 |
| Rodriguez-Laguna 2023 (EULAR poster) | G3 | 5 | NA | NA | NA | NA | NA | >0.05 |
| Cincinelli 2023 (EULAR oral) | G1, G4, G6 | 5 | NA | NA | NA | NA | NA | >0.05 |
| Harrison 2024 (EULAR poster) | G2, G4 | 6 | NA | NA | NA | NA | NA | >0.2 |

Table summarising the results of univariate and multivariate tests for this predictor across all studies included in PICO3. The definitions provided by the authors for D2M/TR PsA are summarised into groups, G1-G6, defined as follows**:** G1=loss/ lack of response to 1st b/tsDMARD; G2=loss/ lack of response to ≥2 b/tsDMARDs, with ≥2 mechanisms of action (MOA); G3=loss/ lack of response to b/tsDMARDs, mechanism and line of treatment not specified; G4=failure to achieve MDA by any composite measure ; G5=objective inflammation (SJC ≥3 OR raised CRP/ESR); G6=Patient/physician perception

**Key:** CI=confidence interval; d=definition, in the event that authors tested different definition of D2M/TR PsA in their paper; HR=hazard ratio; m=refers to different statistical models, in the event that the authors applied multiple statistical models to the same data; NA=not applicable/ missing; NOS ROB=Newcastle-Ottawa Scale Risk of Bias Score; OR=odds ratio; p val=p value; RR=risk ratio.

# **Supplementary Table 15.** Summary of odds/risk/hazard ratio and confidence interval/p values for each study that explored BMI as a predictor of D2M/TR PsA

| **BMI** | | | | | | | | |
| --- | --- | --- | --- | --- | --- | --- | --- | --- |
|  |  |  | **Multivariate** | | | **Univariate** | | |
| **Author and year** | **Group (G1-G6)** | **NOS ROB score** | **OR/RR/HR** | **95% CI** | **p val** | **OR/RR/HR** | **95% CI** | **p val** |
| Harrold 2017 (d1) | G1 | 6 | 1.02 | 0.99-1.05 | >0.05 | NA | NA | NA |
| Harrold 2017 (d2) | G2 | 6 | 0.98 | 0.96-1.01 | >0.05 | NA | NA | NA |
| Iannone 2021 (d1) | G1 | 6 | NA | NA | NA | NA | NA | >0.05 |
| Iannone 2021 (d2) | G2 | 6 | NA | NA | NA | NA | NA | >0.05 |
| Joven 2023 | G3 | 5 | NA | NA | NA | 0.85 | 0.39-1.82 | 0.685 |
| Lorenzin 2021 (d1) | G2 | 6 | 1.02 | 0.98-1.07 | 0.392 | NA | NA | NA |
| Genteloni 2023 | G2, G4 | 5 | NA | NA | <0.01 | NA | NA | NA |
| Perrota 2022 | G1, G4, G6 | 6 | NA | NA | NA | NA | NA | 0.032 |
| Philippoteaux 2023 | G1 | 6 | 1.21 | 0.88-1.64 | 0.23 | NA | NA | NA |
| Rida 2023 (d1) | G3 | 5 | 0.56 | 0.31-1.0 | 0.049 | NA | NA | NA |
| Rida 2023 (d2) | G1 | 5 | NA | NA | <0.05 | NA | NA | NA |
| Rida 2023 (d3) | G2 | 5 | 0.64 | 0.42-1.0 | 0.048 | NA | NA | NA |
| Samuel 2024 (m1) | G6 | 5 | NA | NA | NA | NA | NA | 0.059 |
| Samuel 2024 (m2) | G6 | 5 | 1.06 | 0.98-1.15 | 0.16 | NA | NA | NA |
| Samuel 2024 (m3) | G6 | 5 | 1.06 | 0.97-1.14 | 0.18 | NA | NA | NA |
| Samuel 2024 (m4) | G6 | 5 | 1.04 | 0.96-1.13 | 0.3 | NA | NA | NA |
| Samuel 2024 (m5) | G6 | 5 | 1.08 | 0.99-1.17 | 0.08 | NA | NA | NA |
| Samuel 2024 (m6) | G6 | 5 | 1.07 | 0.99-1.16 | 0.11 | NA | NA | NA |
| Samuel 2024 (m7) | G6 | 5 | 1.05 | 0.97-1.14 | 0.19 | NA | NA | NA |
| Mateo-Soria 2022 | G2 | 5 | NA | NA | NA | 1.01 | 0.94-1.10 | 0.724 |
| Tsurata 2019 | G2 | 6 | 2.04 | 0.94-4.47 | 0.07 | NA | NA | 0.88 |
| Alp 2024 | G1, G4 | 6 | NA | NA | NA | NA | NA | 0.15 |
| Vassilaklis 2024 (d1) | G2, G4 | 6 | 1.07 | 1.01-1.13 | 0.023 | NA | NA | 0.002 |
| Vassilaklis 2024 (d2) | G2, G4 | 6 | NA | NA | >0.05 | NA | NA | 0.004 |
| Vassilaklis 2024 (d3) | G2, G4 | 6 | NA | NA | NA | NA | NA | 0.877 |
| Cincinelli 2023 (EULAR oral) | G1, G4, G6 | 5 | NA | NA | NA | NA | NA | >0.05 |
| Harrison 2024 (EULAR poster) | G2, G4 | 6 | NA | NA | NA | NA | NA | >0.2 |

Table summarising the results of univariate and multivariate tests for this predictor across all studies included in PICO3. The definitions provided by the authors for D2M/TR PsA are summarised into groups, G1-G6, defined as follows**:** G1=loss/ lack of response to 1st b/tsDMARD; G2=loss/ lack of response to ≥2 b/tsDMARDs, with ≥2 mechanisms of action (MOA); G3=loss/ lack of response to b/tsDMARDs, mechanism and line of treatment not specified; G4=failure to achieve MDA by any composite measure ; G5=objective inflammation (SJC ≥3 OR raised CRP/ESR); G6=Patient/physician perception

**Key:** CI=confidence interval; d=definition, in the event that authors tested different definition of D2M/TR PsA in their paper; HR=hazard ratio; m=refers to different statistical models, in the event that the authors applied multiple statistical models to the same data; NA=not applicable/ missing; NOS ROB=Newcastle-Ottawa Scale Risk of Bias Score; OR=odds ratio; p val=p value; RR=risk ratio.

# **Supplementary Table 16.** Summary of odds/risk/hazard ratio and confidence interval/p values for each study that explored csDMARD use as a predictor of D2M/TR PsA

| **csDMARD** | | | | | | | | |
| --- | --- | --- | --- | --- | --- | --- | --- | --- |
|  |  |  | **Multivariate** | | | **Univariate** | | |
| **Author and year** | **Group (G1-G6)** | **NOS ROB score** | **OR/RR/HR** | **95% CI** | **p val** | **OR/RR/HR** | **95% CI** | **p val** |
| Dalen 2023 | G1 | 6 | 1.104 | NA | 0.055 | 1.095 | NA | 0.0727 |
| Glintborg 2011 | G1 | 6 | 1.37 | 1.07-1.75 | 0.013 | 1.21 | 0.98-1.50 | 0.082 |
| Hunter 2022 | G3 | 6 | 2.14 | 1.20-3.55 | 0.0033 | NA | NA | NA |
| Iannone 2021 (d1) | G1 | 6 | NA | NA | NA | NA | NA | >0.05 |
| Iannone 2021 (d2) | G2 | 6 | NA | NA | NA | NA | NA | >0.05 |
| Joven 2023 | G3 | 5 | NA | NA | NA | 1.55 | 0.74-3.26 | 0.249 |
| Roseman 2024 (d1) | G5 | 5 | 0.44 | 0.21-0.92 | <0.05 | 0.37 | 0.19-0.73 | <0.05 |
| Roseman 2024 (d2) | G6 | 5 | 0.45 | 0.19-1.09 | >0.05 | 0.45 | 0.21-0.96 | <0.05 |
| Saad 2009 (d1) | G3 | 6 | NA | NA | >0.05 | NA | NA | >0.05 |
| Mateo-Soria 2022 | G2 | 5 | NA | NA | NA | 1.7 | 0.10-28.2 | 0.709 |
| Stober 2018 (d1) | G2 | 5 | NA | NA | NA | NA | NA | >0.05 |
| Stober 2018 (d2) | G3 | 5 | NA | NA | NA | 0.15 | 0.03-0.74 | 0.02 |
| Alp 2024 | G1, G4 | 6 | NA | NA | NA | NA | NA | >0.05 |
| Vassilaklis 2024 (d1) | G2, G4 | 6 | NA | NA | NA | NA | NA | >0.05 |
| Vassilaklis 2024 (d2) | G2, G4 | 6 | NA | NA | NA | NA | NA | >0.05 |
| Vassilaklis 2024 (d3) | G2, G4 | 6 | NA | NA | NA | NA | NA | >0.05 |
| Rodriguez-Laguna 2023 (EULAR poster) | G3 | 5 | NA | NA | NA | 10.73 | 8.41-14.02 | <0.05 |
| Cincinelli 2023 (EULAR oral) | G1, G4, G6 | 5 | NA | NA | NA | NA | NA | >0.05 |
| Ferreira 2024 (EULAR oral) | G3 | 6 | NA | NA | NA | NA | NA | 0.858 |
| Harrison 2024 (EULAR poster) | G2, G4 | 6 | NA | NA | NA | NA | NA | >0.05 |
| Kalyoncu 2024 (EULAR oral) | G1 | 6 | NA | NA | NA | 1.47 | 1.28-1.68 | <0.05 |

Table summarising the results of univariate and multivariate tests for this predictor across all studies included in PICO3. The definitions provided by the authors for D2M/TR PsA are summarised into groups, G1-G6, defined as follows**:** G1=loss/ lack of response to 1st b/tsDMARD; G2=loss/ lack of response to ≥2 b/tsDMARDs, with ≥2 mechanisms of action (MOA); G3=loss/ lack of response to b/tsDMARDs, mechanism and line of treatment not specified; G4=failure to achieve MDA by any composite measure ; G5=objective inflammation (SJC ≥3 OR raised CRP/ESR); G6=Patient/physician perception

**Key:** CI=confidence interval; d=definition, in the event that authors tested different definition of D2M/TR PsA in their paper; HR=hazard ratio; m=refers to different statistical models, in the event that the authors applied multiple statistical models to the same data; NA=not applicable/ missing; NOS ROB=Newcastle-Ottawa Scale Risk of Bias Score; OR=odds ratio; p val=p value; RR=risk ratio.

# **Supplementary Table 17.** Summary of odds/risk/hazard ratio and confidence interval/p values for each study that explored skin psoriasis as a predictor of D2M/TR PsA

| **PsO skin** | | | | | | | | |
| --- | --- | --- | --- | --- | --- | --- | --- | --- |
|  |  |  | **Multivariate** | | | **Univariate** | | |
| **Author and year** | **Group (G1-G6)** | **NOS ROB score** | **OR/RR/HR** | **95% CI** | **p val** | **OR/RR/HR** | **95% CI** | **p val** |
| Harrold 2017 (d1) | G1 | 6 | 1.18 | 0.74-1.87 | >0.05 | NA | NA | NA |
| Harrold 2017 (d2) | G2 | 6 | 1.38 | 1.01-1.87 | <0.05 | NA | NA | NA |
| Lorenzin 2021 (d1) | G2 | 6 | 0.95 | 0.86-1.04 | 0.263 | NA | NA | NA |
| Genteloni 2023 | G2, G4 | 5 | NA | NA | 0.203 | 2.16 | 0.88-5.32 | 0.092 |
| Perrota 2022 | G1, G4, G6 | 6 | NA | NA | NA | NA | NA | <0.001/ <0.01 |
| Samuel 2024 (m1) | G6 | 5 | 0.854 | 0.756-0.965 | 0.011 | NA | NA | <0.001 |
| Samuel 2024 (m2) | G6 | 5 | 0.85 | 0.71-1.02 | 0.08 | NA | NA | NA |
| Samuel 2024 (m3) | G6 | 5 | 0.85 | 0.71-1.01 | 0.05 | NA | NA | NA |
| Samuel 2024 (m4) | G6 | 5 | 0.85 | 0.71-1.01 | 0.06 | NA | NA | NA |
| Samuel 2024 (m5) | G6 | 5 | 0.85 | 0.71-1.02 | 0.08 | NA | NA | NA |
| Samuel 2024 (m6) | G6 | 5 | 0.82 | 0.68-0.99 | 0.04 | NA | NA | NA |
| Samuel 2024 (m7) | G6 | 5 | 0.83 | 0.69-1 | 0.05 | NA | NA | NA |
| Tsurata 2019 | G2 | 6 | 1.25 | 0.58-2.69 | 0.57 | NA | NA | 0.01 |
| Alp 2024 | G1, G4 | 6 | NA | NA | >0.05 | NA | NA | 0.038 |
| Vassilaklis 2024 (d1) | G2, G4 | 6 | NA | NA | NA | NA | NA | <0.05 |
| Vassilaklis 2024 (d2) | G2, G4 | 6 | NA | NA | NA | NA | NA | <0.05 |
| Vassilaklis 2024 (d3) | G2, G4 | 6 | NA | NA | NA | NA | NA | <0.05 |
| Rodriguez-Laguna 2023 (EULAR poster) | G3 | 5 | NA | NA | NA | 11.04 | 8.67-14.02 | <0.05 |
| Cincinelli 2023 (EULAR oral) | G1, G4, G6 | 5 | NA | NA | NA | NA | NA | >0.05 |

Table summarising the results of univariate and multivariate tests for this predictor across all studies included in PICO3. The definitions provided by the authors for D2M/TR PsA are summarised into groups, G1-G6, defined as follows**:** G1=loss/ lack of response to 1st b/tsDMARD; G2=loss/ lack of response to ≥2 b/tsDMARDs, with ≥2 mechanisms of action (MOA); G3=loss/ lack of response to b/tsDMARDs, mechanism and line of treatment not specified; G4=failure to achieve MDA by any composite measure ; G5=objective inflammation (SJC ≥3 OR raised CRP/ESR); G6=Patient/physician perception

**Key:** CI=confidence interval; d=definition, in the event that authors tested different definition of D2M/TR PsA in their paper; HR=hazard ratio; m=refers to different statistical models, in the event that the authors applied multiple statistical models to the same data; NA=not applicable/ missing; NOS ROB=Newcastle-Ottawa Scale Risk of Bias Score; OR=odds ratio; p val=p value; RR=risk ratio.

# **Supplementary Table 18**. Summary of odds/risk/hazard ratio and confidence interval/p values for each study that explored dactylitis as a predictor of D2M/TR PsA

| **Dactylitis** | | | | | | | | |
| --- | --- | --- | --- | --- | --- | --- | --- | --- |
|  |  |  | **Multivariate** | | | **Univariate** | | |
| **Author and year** | **Group (G1-G6)** | **NOS ROB score** | **OR/RR/HR** | **95% CI** | **p val** | **OR/RR/HR** | **95% CI** | **p val** |
| Perrota 2022 | G1, G4, G6 | 6 | NA | NA | NA | NA | NA | 0.873 |
| Samuel 2024 (m1) | G6 | 5 | 0.226 | 0.042-1.213 | 0.083 | NA | NA | 0.33 |
| Samuel 2024 (m2) | G6 | 5 | 2.61 | 0.27-25.64 | 0.41 | NA | NA | NA |
| Samuel 2024 (m3) | G6 | 5 | 3.55 | 0.41-30.45 | 0.25 | NA | NA | NA |
| Samuel 2024 (m4) | G6 | 5 | 3.11 | 0.35-27.52 | 0.31 | NA | NA | NA |
| Samuel 2024 (m5) | G6 | 5 | 1.45 | 0.15-13.99 | 0.75 | NA | NA | NA |
| Samuel 2024 (m6) | G6 | 5 | 2.07 | 0.25-16.91 | 0.5 | NA | NA | NA |
| Samuel 2024 (m7) | G6 | 5 | 1.67 | 0.21-13.40 | 0.63 | NA | NA | NA |
| Mateo-Soria 2022 | G2 | 5 | NA | NA | NA | 1.13 | 0.48-2.66 | 0.783 |
| Tsurata 2019 | G2 | 6 | NA | NA | NA | NA | NA | 0.86 |
| Alp 2024 | G1, G4 | 6 | NA | NA | NA | NA | NA | 0.278 |
| Vassilaklis 2024 (d1) | G2, G4 | 6 | NA | NA | NA | NA | NA | 0.793 |
| Additional data |  | 6 | NA | NA | >0.5 | NA | NA | 0.048 |
| Vassilaklis 2024 (d2) | G2, G4 | 6 | NA | NA | NA | NA | NA | 0.38 |
| Additional data |  | 6 | NA | NA | NA | NA | NA | 0.476 |
| Vassilaklis 2024 (d3) | G2, G4 | 6 | NA | NA | NA | NA | NA | 0.876 |
| Additional data |  | 6 | NA | NA | NA | NA | NA | 0.108 |
| Rodriguez-Laguna 2023 (EULAR poster) | G3 | 5 | NA | NA | NA | 49.54 | 23.61-103.91 | <0.05 |
| Cincinelli 2023 (EULAR oral) | G1, G4, G6 | 5 | NA | NA | NA | NA | NA | >0.05 |
| Ferreira 2024 (EULAR oral) | G3 | 6 | NA | NA | NA | NA | NA | 0.468/0.683 |
| Harrison 2024 (EULAR poster) | G2, G4 | 6 | NA | NA | NA | NA | NA | >0.200 |

Table summarising the results of univariate and multivariate tests for this predictor across all studies included in PICO3. The definitions provided by the authors for D2M/TR PsA are summarised into groups, G1-G6, defined as follows**:** G1=loss/ lack of response to 1st b/tsDMARD; G2=loss/ lack of response to ≥2 b/tsDMARDs, with ≥2 mechanisms of action (MOA); G3=loss/ lack of response to b/tsDMARDs, mechanism and line of treatment not specified; G4=failure to achieve MDA by any composite measure ; G5=objective inflammation (SJC ≥3 OR raised CRP/ESR); G6=Patient/physician perception

**Key:** CI=confidence interval; d=definition, in the event that authors tested different definition of D2M/TR PsA in their paper; HR=hazard ratio; m=refers to different statistical models, in the event that the authors applied multiple statistical models to the same data; NA=not applicable/ missing; NOS ROB=Newcastle-Ottawa Scale Risk of Bias Score; OR=odds ratio; p val=p value; RR=risk ratio.

# **Supplementary Table 19.** Summary of odds/risk/hazard ratio and confidence interval/p values for each study that explored swollen joint count as a predictor of D2M/TR PsA

| **SJC** | | | | | | | | |
| --- | --- | --- | --- | --- | --- | --- | --- | --- |
|  |  |  | **Multivariate** | | | **Univariate** | | |
| **Author and year** | **Group (G1-G6)** | **NOS ROB score** | **OR/RR/HR** | **95% CI** | **p val** | **OR/RR/HR** | **95% CI** | **p val** |
| Glintborg 2011 | G1 | 6 | NA | NA | NA | 0.99 | 0.97-1.02 | 0.59 |
| Rida 2023 (d1) | G3 | 5 | NA | NA | <0.05 | NA | NA | NA |
| Rida 2023 (d2) | G1 | 5 | 1.14 | 1.03-1.25 | 0.008 | NA | NA | NA |
| Rida 2023 (d3) | G2 | 5 | NA | NA | >0.05 | NA | NA | NA |
| Roseman 2024 (d1) | G5 | 5 | NA | NA |  | 0.96 | 0.90-1.02 | 0.15 |
| Roseman 2024 (d2) | G6 | 5 | NA | 0.83-0.99 | 0.03 | 0.91 | 0.84-0.99 | <0.05 |
| Saad 2009 (d1) | G3 | 6 | 0.97 | 0.96-0.99 | <0.05 | NA | NA | NA |
| Samuel 2024 (m1) | G6 | 5 | 0.827 | 0.745-0.918 | <0.001 | NA | NA | <0.001 |
| Samuel 2024 (m2) | G6 | 5 | 0.77 | 0.60-0.99 | 0.04 | NA | NA | NA |
| Samuel 2024 (m3) | G6 | 5 | 0.75 | 0.59-0.96 | 0.02 | NA | NA | NA |
| Samuel 2024 (m4) | G6 | 5 | 0.74 | 0.59-0.94 | 0.01 | NA | NA | NA |
| Mateo-Soria 2022 | G2 | 5 | 1.53 | 1.07-2.20 | 0.021 | 1.52 | 1.11-2.09 | 0.009 |
| Stober 2018 (d1) | G2 | 5 | NA | NA | NA | 1.01 | 0.99-1.02 | 0.36 |
| Stober 2018 (d2) | G3 | 5 | NA | NA | NA | 1.06 | 0.99-1.13 | 0.07 |
| Alp 2024 | G1, G4 | 6 | NA | NA | >0.05 | NA | NA | <0.001 |
| Ferreira 2024 (EULAR oral) | G3 | 6 | NA | NA | NA | NA | 0.112/0.105 | >0.05 |
| Harrison 2024 (EULAR poster) | G2, G4 | 6 | NA | NA | NA | NA | NA | >0.200 |

Table summarising the results of univariate and multivariate tests for this predictor across all studies included in PICO3. The definitions provided by the authors for D2M/TR PsA are summarised into groups, G1-G6, defined as follows**:** G1=loss/ lack of response to 1st b/tsDMARD; G2=loss/ lack of response to ≥2 b/tsDMARDs, with ≥2 mechanisms of action (MOA); G3=loss/ lack of response to b/tsDMARDs, mechanism and line of treatment not specified; G4=failure to achieve MDA by any composite measure ; G5=objective inflammation (SJC ≥3 OR raised CRP/ESR); G6=Patient/physician perception

**Key:** CI=confidence interval; d=definition, in the event that authors tested different definition of D2M/TR PsA in their paper; HR=hazard ratio; m=refers to different statistical models, in the event that the authors applied multiple statistical models to the same data; NA=not applicable/ missing; NOS ROB=Newcastle-Ottawa Scale Risk of Bias Score; OR=odds ratio; p val=p value; RR=risk ratio.

# **Supplementary Table 20.** Summary of odds/risk/hazard ratio and confidence interval/p values for each study that explored PsA duration as a predictor of D2M/TR PsA

| **PsA duration (years)** | | | | | | | | |
| --- | --- | --- | --- | --- | --- | --- | --- | --- |
|  |  |  | **Multivariate** | | | **Univariate** | | |
| **Author and year** | **Group (G1-G6)** | **NOS ROB score** | **OR/RR/HR** | **95% CI** | **p val** | **OR/RR/HR** | **95% CI** | **p val** |
| Harrold 2017 (d1) | G1 | 6 | 0.96 | 0.92-0.99 | <0.05 | NA | NA | NA |
| Harrold 2017 (d2) | G2 | 6 | 0.99 | 0.97-1.00 | <0.05 | NA | NA | NA |
| Iannone 2021 (d1) | G1 | 6 | NA | NA | NA | NA | NA | >0.05 |
| Iannone 2021 (d2) | G2 | 6 | NA | NA | NA | NA | NA | <0.01 |
| Perrota 2022 | G1, G4, G6 | 6 | NA | NA | NA | NA | NA | 0.668 |
| Roseman 2024 (d1) | G5 | 5 | NA | NA | NA | 1.01 | 0.97-1.04 | 0.79 |
| Roseman 2024 (d2) | G6 | 5 | NA | NA | NA | 0.97 | 0.93-1.01 | 0.18 |
| Samuel 2024 (m1) | G6 | 5 | NA | NA | NA | NA | NA | 0.69 |
| Vieira-Sousa 2020 | G3 | 6 | 1 | 0.97-1.03 | 0.91 | NA | NA | NA |
| Mateo-Soria 2022 | G2 | 5 | NA | NA | NA | 1.01 | 0.97-1.05 | 0.312 |
| Stober 2018 (d1) | G2 | 5 | NA | NA | NA | 0.93 | 0.88-0.99 | 0.02 |
| Stober 2018 (d2) | G3 | 5 | NA | NA | NA | 1.01 | 0.91-1.12 | 0.87 |
| Tsurata 2019 | G2 | 6 | NA | NA | NA | NA | NA | 0.06 |
| Alp 2024 | G1, G4 | 6 | NA | NA | >0.05 | NA | NA | 0.019 |
| Vassilaklis 2024 (d1) | G2, G4 | 6 | NA | NA | NA | NA | NA | 0.792 |
| Vassilaklis 2024 (d2) | G2, G4 | 6 | NA | NA | NA | NA | NA | 0.55 |
| Vassilaklis 2024 (d3) | G2, G4 | 6 | NA | NA | NA | NA | NA | 0.499 |
| Cincinelli 2023 (EULAR oral) | G1, G4, G6 | 5 | NA | NA | NA | NA | NA | >0.05 |
| Harrison 2024 (EULAR poster) | G2, G4 | 6 | NA | NA | NA | NA | NA | >0.2 |

Table summarising the results of univariate and multivariate tests for this predictor across all studies included in PICO3. The definitions provided by the authors for D2M/TR PsA are summarised into groups, G1-G6, defined as follows**:** G1=loss/ lack of response to 1st b/tsDMARD; G2=loss/ lack of response to ≥2 b/tsDMARDs, with ≥2 mechanisms of action (MOA); G3=loss/ lack of response to b/tsDMARDs, mechanism and line of treatment not specified; G4=failure to achieve MDA by any composite measure ; G5=objective inflammation (SJC ≥3 OR raised CRP/ESR); G6=Patient/physician perception

**Key:** CI=confidence interval; d=definition, in the event that authors tested different definition of D2M/TR PsA in their paper; HR=hazard ratio; m=refers to different statistical models, in the event that the authors applied multiple statistical models to the same data; NA=not applicable/ missing; NOS ROB=Newcastle-Ottawa Scale Risk of Bias Score; OR=odds ratio; p val=p value; RR=risk ratio.

# **Supplementary Table 21**. Summary of odds/risk/hazard ratio and confidence interval/p values for each study that explored tender joint count as a predictor of D2M/TR PsA

| **TJC** | | | | | | | | |
| --- | --- | --- | --- | --- | --- | --- | --- | --- |
|  |  |  | **Multivariate** | | | **Univariate** | | |
| **Author and year** | **Group (G1-G6)** | **NOS ROB score** | **OR/RR/HR** | **95% CI** | **p val** | **OR/RR/HR** | **95% CI** | **p val** |
| Glintborg 2011 | G1 | 6 | NA | NA | NA | 1.03 | 1.02-1.05 | <0.001 |
| Rida 2023 (d1) | G3 | 5 | NA | NA | <0.05 | NA | NA | NA |
| Rida 2023 (d2) | G1 | 5 | 1.06 | 1.02-1.1 | 0.002 | NA | NA | NA |
| Rida 2023 (d3) | G2 | 5 | 1.04 | 1.01-1.07 | 0.019 | NA | NA | NA |
| Roseman 2024 (d1) | G5 | 5 | NA | NA | NA | 1 | 0.96-1.05 | 0.9 |
| Roseman 2024 (d2) | G6 | 5 | NA | NA | NA | 0.99 | 0.93-1.04 | 0.65 |
| Saad 2009 (d1) | G3 | 6 | 1 | 0.99-1.01 | >0.05 | NA | NA | NA |
| Samuel 2024 (m1) | G6 | 5 | 0.861 | 0.803-0.923 | <0.001 | NA | NA | <0.001 |
| Samuel 2024 (m5) | G6 | 5 | 0.85 | 0.72-1.01 | 0.06 | NA | NA | NA |
| Samuel 2024 (m6) | G6 | 5 | 0.83 | 0.70-0.98 | 0.03 | NA | NA | NA |
| Samuel 2024 (m7) | G6 | 5 | 0.83 | 0.70-0.97 | 0.02 | NA | NA | NA |
| Mateo-Soria 2022 | G2 | 5 | NA | NA | NA | 1.29 | 1.29-1.59 | 0.018 |
| Stober 2018 (d1) | G2 | 5 | NA | NA | NA | 1.01 | 0.99-1.02 | 0.36 |
| Stober 2018 (d2) | G3 | 5 | NA | NA | NA | 1 | 0.97-1.04 | 0.81 |
| Alp 2024 | G1, G4 | 6 | NA | NA | >0.05 | NA | NA | <0.001 |
| Ferreira 2024 (EULAR oral) | G3 | 6 | NA | NA | NA | NA | 0.198/0.176 | >0.05 |
| Harrison 2024 (EULAR poster) | G2, G4 | 6 | NA | NA | NA | NA | NA | >0.200 |

Table summarising the results of univariate and multivariate tests for this predictor across all studies included in PICO3. The definitions provided by the authors for D2M/TR PsA are summarised into groups, G1-G6, defined as follows**:** G1=loss/ lack of response to 1st b/tsDMARD; G2=loss/ lack of response to ≥2 b/tsDMARDs, with ≥2 mechanisms of action (MOA); G3=loss/ lack of response to b/tsDMARDs, mechanism and line of treatment not specified; G4=failure to achieve MDA by any composite measure ; G5=objective inflammation (SJC ≥3 OR raised CRP/ESR); G6=Patient/physician perception

**Key:** CI=confidence interval; d=definition, in the event that authors tested different definition of D2M/TR PsA in their paper; HR=hazard ratio; m=refers to different statistical models, in the event that the authors applied multiple statistical models to the same data; NA=not applicable/ missing; NOS ROB=Newcastle-Ottawa Scale Risk of Bias Score; OR=odds ratio; p val=p value; RR=risk ratio.

# **Supplementary Table 22.** Summary of odds/risk/hazard ratio and confidence interval/p values for each study that explored enthesitis as a predictor of D2M/TR PsA

| **Enthesitis*** | | | | | | | | |
| --- | --- | --- | --- | --- | --- | --- | --- | --- |
|  |  |  | **Multivariate** | | | **Univariate** | | |
| **Author and year** | **Group (G1-G6)** | **NOS ROB score** | **OR/RR/HR** | **95% CI** | **p val** | **OR/RR/HR** | **95% CI** | **p val** |
| Samuel 2024 (m1) | G6 | 5 | 0.208 | 0.061-0.717 | 0.013 | NA | NA | 0.04/0.001 |
| Samuel 2024 (m2) | G6 | 5 | 1.78 | 0.48-6.59 | 0.39 | NA | NA | NA |
| Samuel 2024 (m3) | G6 | 5 | 1.16 | 0.27-4.96 | 0.84 | NA | NA | NA |
| Samuel 2024 (m4) | G6 | 5 | 1.38 | 0.39-4.92 | 0.62 | NA | NA | NA |
| Samuel 2024 (m5) | G6 | 5 | 2.75 | 0.64-11.76 | 0.17 | NA | NA | NA |
| Samuel 2024 (m6) | G6 | 5 | 1.78 | 0.41-7.62 | 0.44 | NA | NA | NA |
| Samuel 2024 (m7) | G6 | 5 | 2.22 | 0.53-9.26 | 0.28 | NA | NA | NA |
| Tsurata 2019 | G2 | 6 | NA | NA | NA | NA | NA | 0.57 |
| Alp 2024 | G1, G4 | 6 | NA | NA | NA | NA | NA | 0.425/ 0.751 |
| Vassilaklis 2024 (d1) | G2, G4 | 6 | NA | NA | NA | NA | NA | 0.166 |
| Additional data |  | 6 | NA | NA | NA | NA | NA | 0.103 |
| Vassilaklis 2024 (d2) | G2, G4 | 6 | NA | NA | NA | NA | NA | 0.093 |
| Additional data |  | 6 | NA | NA | NA | NA | NA | 0.535 |
| Vassilaklis 2024 (d3) | G2, G4 | 6 | NA | NA | NA | NA | NA | 0.289 |
| Additional data |  | 6 | NA | NA | NA | NA | NA | 0.325 |
| Rodriguez-Laguna 2023 (EULAR poster) | G3 | 5 | NA | NA | NA | 30.8 | 18.87-50.28 | <0.05 |
| Cincinelli 2023 (EULAR oral) | G1, G4, G6 | 5 | NA | NA | NA | NA | NA | >0.05 |
| Ferreira 2024 (EULAR oral) | G3 | 6 | NA | NA | NA | NA | NA | 0.577/0.537 |
| Harrison 2024 (EULAR poster) | G2, G4 | 6 | NA | NA | 0.038 | NA | NA | <0.001 |

Table summarising the results of univariate and multivariate tests for this predictor across all studies included in PICO3. The definitions provided by the authors for D2M/TR PsA are summarised into groups, G1-G6, defined as follows**:** G1=loss/ lack of response to 1st b/tsDMARD; G2=loss/ lack of response to ≥2 b/tsDMARDs, with ≥2 mechanisms of action (MOA); G3=loss/ lack of response to b/tsDMARDs, mechanism and line of treatment not specified; G4=failure to achieve MDA by any composite measure ; G5=objective inflammation (SJC ≥3 OR raised CRP/ESR); G6=Patient/physician perception

**Key:** CI=confidence interval; d=definition, in the event that authors tested different definition of D2M/TR PsA in their paper; HR=hazard ratio; m=refers to different statistical models, in the event that the authors applied multiple statistical models to the same data; NA=not applicable/ missing; NOS ROB=Newcastle-Ottawa Scale Risk of Bias Score; OR=odds ratio; p val=p value; RR=risk ratio.
